# Trioxidized cysteine in the aging proteome mimics the structural dynamics and interactome of phosphorylated serine

**DOI:** 10.1111/acel.14062

**Published:** 2023-12-18

**Authors:** Jose Antonio Sánchez Milán, María Fernández‐Rhodes, Xue Guo, María Mulet, SoFong Cam Ngan, Ranjith Iyappan, Maryam Katoueezadeh, Siu Kwan Sze, Aida Serra, Xavier Gallart‐Palau

**Affiliations:** ^1^ Biomedical Research Institute of Lleida (IRBLLEIDA) ‐ +Pec Proteomics Research Group (+PPRG) ‐ Neuroscience Area University Hospital Arnau de Vilanova (HUAV) Lleida Spain; ^2^ Department of Basic Medical Sciences, Biomedical Research Institute of Lleida (IRB Lleida) ‐ +Pec Proteomics Research Group (+PPRG) ‐ Neuroscience Area University of Lleida (UdL) Lleida Spain; ^3^ Institute of Molecular and Cell Biology (IMCB) Singapore Singapore; ^4^ Department of Health Sciences, Faculty of Applied Health Sciences Brock University St. Catharines Ontario Canada; ^5^ Department of Psychology University of Lleida (UdL) Lleida Spain

**Keywords:** aging, bioinformatics, degenerative protein modifications, kinases, molecular dynamics, phosphorylation, post‐translational modifications, protein structure, proteome, signaling, thiol trioxidized cysteine

## Abstract

Aging is the primary risk factor for the development of numerous human chronic diseases. On a molecular level, it significantly impacts the regulation of protein modifications, leading to the accumulation of degenerative protein modifications (DPMs) such as aberrant serine phosphorylation (p‐Ser) and trioxidized cysteine (t‐Cys) within the proteome. The altered p‐Ser is linked to abnormal cell signaling, while the accumulation of t‐Cys is associated with chronic diseases induced by oxidative stress. Despite this, the potential cross‐effects and functional interplay between these two critical molecular factors of aging remain undisclosed. This study analyzes the aging proteome of wild‐type C57BL/6NTac mice over 2 years using advanced proteomics and bioinformatics. Our objective is to provide a comprehensive analysis of how t‐Cys affects cell signaling and protein structure in the aging process. The results obtained indicate that t‐Cys residues accumulate in the aging proteome, interact with p‐Ser interacting enzymes, as validated in vitro, and alter their structures similarly to p‐Ser. These findings have significant implications for understanding the interplay of oxidative stress and phosphorylation in the aging process. Additionally, they open new venues for further research on the role(s) of these protein modifications in various human chronic diseases and aging, wherein exacerbated oxidation and aberrant phosphorylation are implicated.

## Graphical abstract

Trioxidized cysteine, product of cellular prooxidant processes, demonstrates an age‐related increase in various mammalian proteomes. These post‐translationally modified residues exhibit the capacity to interact with enzymes that mediate phosphorylation signaling, mimicking features of phosphorylated serine residues and likely contributing to the alteration of cell signaling during aging. Additionally, we demonstrate that phosphorylation‐linked kinase activity becomes modulated in an oxidation‐dependent manner.

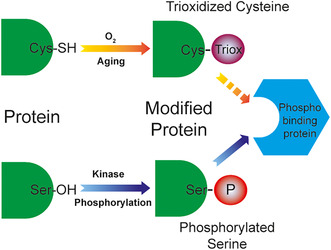

**Abbreviations**
AA: ammonium acetate
ABB: ammonium bicarbonate
ACN: acetonitrile
Asp: asparagine
BCA: bicinchoninic acid assay
DPMs: degenerative protein modifications
FA: formic acid
Gln: glutamine
HCD: high‐energy collisional dissociation
HPLC: high‐pressure liquid chromatography
LC–MS/MS: liquid chromatography coupled tandem mass spectrometry
Met: metionine
p‐Ser: phosphoserine
PTB: phosphotyrosine binding domain
PTMs: post‐translational modifications
SDS: sodium dodecyl sulphate
SDS‐PAGE: sodium dodecyl sulfate polyacrylamide gel electrophoresis
SH2: Src Homology 2
t‐Cys: trioxidized cysteine
Thr: threonine
Tyr: tyrosine

## 1 INTRODUCTION

Post‐translational modifications (PTMs) are molecular decorations, products of spontaneous or enzymatic reactions, that regulate the conformation and molecular dynamics of proteins (Jennings et al., 2022). Currently, over 700 PTMs have been identified, which significantly extend the proteome diversity of the organism (Jennings et al., 2022; Kalailingam et al., 2023). Phosphorylation is the most relevant PTM that plays a crucial role in regulating various cellular processes, including genome stability, proteostasis, cellular signaling, and cell metabolism (Hepowit et al., 2022; Johnson & Barford, 1993; Roy et al., 2023; Tarrant & Cole, 2009). This modification is facilitated by a family of enzymes known as protein kinases, which catalyze the addition of phosphate groups from ATP molecules to the specific protein amino acid residues serine (Ser), threonine (Thr), and tyrosine (Tyr; Audagnotto & Dal Peraro, 2017; Cohen, 2002; Mandell et al., 2007). This PTM plays a crucial role in the proper transmission of signal transduction cascades, where a series of phosphorylation events occur sequentially, propagating signals to the nucleus (Stock et al., 2000; Whitmarsh & Davis, 1999). Indeed, these transduction cascades can be initiated either intracellularly or extracellularly, the latter typically being a result of the effects caused by growth factors or hormones (Lin et al., 1999; Wang et al., 2015).

Phosphorylation of Ser (p‐Ser) introduces a negatively charged phosphate group to the primary structure of proteins (Thorsness & Koshland Jr., 1987). This negative charge plays a crucial role in mediating protein folding and interactions (Swann et al., 1999). Proteins containing phospho‐specific binding domains, such as Src Homology 2 (SH2) or Phosphotyrosine binding (PTB) domains, can recognize and bind to the negatively charged p‐Ser, forming signaling complexes (Sawyer, 1998; Schlessinger & Lemmon, 2003). Certainly, these complexes form well‐orchestrated signaling cascades within and throughout the cells. However, aging‐linked destabilizing factors, such as oxidative stress, have the ability to alter this fragile signaling equilibrium (López‐Otín et al., 2013; Salminen & Kaarniranta, 2009; Zhang et al., 2016). For example, an increase in p‐Ser residues affecting redox enzymes is known to activate the antioxidant capacity of these crucial molecules in aging. This increase occurs in response to elevated cellular oxidative stress (Judge et al., 2005; Tsang et al., 2014).

Intriguingly, while it is widely known that aging is associated with an increase in oxidative stress in cells, the gradual damage to the thiol (SH) group of cysteine (Cys) residues by oxidation in the cellular proteome has been largely overlooked (Garrido Ruiz et al., 2022). This sulfhydryl group undergoes modifications in response to gradually increasing oxidative stress, transitioning from monooxidation to dioxidation, and eventually undergoing trioxidation (Paulech et al., 2015). While monooxidation and dioxidation of the sulfhydryl group of Cys are reversible modifications, trioxidized cysteine (t‐Cys) leads to the irreversible generation of sulfonic acid (Cys‐SO_3_H; Paramasivan et al., 2020; Paulech et al., 2015). Therefore, t‐Cys is considered a degenerative protein modification (DPM) linked to tissue degeneration. The effects of t‐Cys have been associated with poorly understood proteinopathy and natural aging, as previously documented by us and other colleagues (Gallart‐Palau et al., 2015; Garner & Spector, 1980; Garrido Ruiz et al., 2022; Guo et al., 2020; Paramasivan et al., 2020; Paulech et al., 2015; Pérez, Bokov, et al., 2009). However, to the best of our knowledge, the molecular dynamic effects of t‐Cys on protein structure and the potential for this post‐translationally modified residue to interfere with cellular protein signaling, due to its nearly identical chemical structure to p‐Ser, have been disregarded until now.

In this work, we used advanced discovery‐driven proteomics to investigate the occurrence of t‐Cys on the proteome of different mouse cohorts alongside the aging process. Additionally, we analyze the occurrence of p‐Ser in these aging‐linked proteomes and perform advanced bioinformatics to decipher any potential effects of t‐Cys on the structure and cell signaling capacity of the aging‐dependent post‐translationally modified proteins. The groundbreaking findings obtained provide compelling evidence, for the first time, that t‐Cys possesses the ability to disrupt the homeostatic equilibrium of the target proteins and impact cellular signaling mechanisms in a manner highly similar to what p‐Ser does. Therefore, t‐Cys exerts a significant and yet undetermined influence on the process of systemic aging.

## 2 MATERIALS AND METHODS

### 2.1 Chemicals and reagents

All chemical reagents and solvents were purchased from Sigma‐Aldrich unless otherwise specified. Sequencing‐grade modified trypsin was purchased from Promega.

### 2.2 Animals

Wild‐type (C57BL/6NTac) mice were housed in cages on a 12‐h dark/light cycle at stable temperature (21°C) with water provided ad libitum and were fed with standard commercial chow. Four different groups of experimental aging were used, including: (i) 1‐month‐old mice; (ii) 6‐month‐old mice; (iii) 18‐month‐old mice; and (iv) 24‐month‐old mice (*n* = 24; 12 male/ 12 female). The minimization of animal suffering was of utmost importance for the authors, and it was taken into careful consideration in all detailed experimental procedures. The animals were deeply anesthetized and subsequently euthanized by cervical dislocation. In line with recent findings (Tsitsipatis et al., 2022), skin samples were deemed optimal for conducting a comprehensive proteome‐level analysis of cellular aging in a specific tissue. Therefore, these samples were collected from the dorsal region of post‐mortem mice, the fur was thoroughly removed prior to being washed in 1X PBS and stored at −80°C till further use. All animal experiments were conducted in a humane manner and were approved by the Institutional Animal Care and Use Committee at Nanyang Technological University in Singapore (IACUC protocol # ARF‐SBS/NIE/LKC‐A18016) or the Animal Care Committee at Brock University (AUP # 22‐08‐04). The experiments strictly adhered to the international guiding principles for animal research and followed guidelines on the care and use of laboratory animals for scientific purposes.

### 2.3 Preparation of skin tissue samples for next‐generation proteomics

#### 2.3.1 Proteome extraction

Skin tissues from all mice were homogenized as previously described (Guo et al., 2020). Briefly, ~50 mg of skin tissue were homogenized in lysis buffer containing 2% sodium deoxycholate (SDC) in 100 mM ammonium acetate (AA) at 4°C using a mixture of beads ranging from 0.9 to 2.0 mm in a bullet blender homogenizer (Next Advance, Inc). All homogenization procedures were strictly conducted at 4°C and, when possible, under a nitrogen atmosphere to prevent any artefactual oxidation, as previously indicated (Giblin et al., 2013). The homogenates were subsequently centrifuged for 10 min at 10,000 × *g* and supernatants were collected. The generated pellet was further homogenized till no remaining pellet was observed and the obtained supernatants were combined. Proteins in the supernatant were acetone precipitated and then quantified by bicinchoninic acid protein assay (BCA) (Thermo).

#### 2.3.2 In‐solution digestion of extracted proteomes

In‐solution tryptic digestion of skin proteomes was performed as we previously described (Lorca, Laparra, et al., 2022; Lorca, Mulet, et al., 2022; Park et al., 2019; Serra et al., 2018, 2016), with minor modifications. Briefly, skin proteomes were diluted five times in 100 mM AA. Reduction was performed with 10 mM DTT in 25 mM AA at 60°C for 30 min, alkylation was performed with 55 mM iodoacetamide in 25 mM AA for 30 min at room temperature. The samples were then further diluted with 100 mM AA to lower the concentration of SDC to 0.5% prior to perform overnight digestion of proteins at 37°C with sequencing‐grade modified trypsin at a trypsin‐to‐protein ratio of 1:50 (w/w). Precipitation of SDC was performed by subjecting the proteome samples to acidic conditions by adding 10% formic acid (FA). Precipitated SDC salts were pelleted by centrifugation (12,000 × *g*; 20 min; room temperature), the supernatants containing the peptides were then collected, and the pellets were resuspended and subjected to acidic precipitation during three additional rounds. All proteome supernatants were collected, combined, and subjected to additional desalting by using C‐18 Sep‐pack 100 mg cartridges (Waters) and subsequently dried in a vacuum concentrator (Eppendorf).

#### 2.3.3 Next‐generation proteomics by liquid chromatography‐tandem mass spectrometry

The obtained proteomes were initially simplified through high‐pressure liquid chromatography fractionation, following the previously indicated procedure without any modifications (Gallart‐Palau et al., 2020). Peptides were then reconstituted in 3% ACN, 0.1% FA before being subjected to liquid chromatography coupled with tandem mass spectrometry (LC–MS/MS) using a Dionex Ultimate 3000 RSLCnano system coupled to Q‐Exactive tandem mass spectrometry (Thermo Fisher Scientific) as detailed previously (Guo et al., 2020). Peptide mixtures (~1 μg/μL) were separated at a flow rate of 300 nL/min using a reverse‐phase Dionex EASY‐Spray PepMap C18 3 μm, 75 μm × 10 cm column (Thermo Fisher Scientific Inc.) maintained at 35°C. Separation was performed in a 60‐min gradient using mobile phase A [0.1% FA in HPLC] and mobile phase B [0.1% FA in ACN] as follows: 3–35% B in 40 min, 35–50% B in 7 min, 50–80% B in 1 min, and 80% B for 2 min, followed by a re‐equilibration of 10 min at 3% B.

Peptides were ionized at an electro‐spray potential of 1.5 kV. For data acquisition in positive mode, a full MS scan was performed with 350–1600 m/z range, resolution 70,000 [at 200 m/z], and maximum ion accumulation time of 100 ms. The 10 most intense ions with a threshold of 2000 counts were fragmented by high energy collisional dissociation (HCD) mode using a normalized collision energy of 28% and a maximum ion accumulation time of 100 ms. The isolation width of 2 was used for MS2.

### 2.4 Bioinformatics and data analysis

#### 2.4.1 Proteomics data from an additional aging mice cohort

To validate the proteomics results generated in this study, we searched the available datasets in ProteomeXchange‐PRIDE repository that analyzed specific proteomes using next‐generation discovery‐driven proteomics in an alternative aged mice cohort. Searching for these criteria, we identified the dataset deposited by (Angelidis et al., 2019) with identifier PXD012307, which includes LC–MS/MS proteomics data of lung tissues from female and male C57BL/6 mice in the following groups: 3, 22, and 24 months. The LC–MS/MS analysis of tissue‐digested proteomes was performed using a Quadrupole/Orbitrap type Mass Spectrometer (Q‐Exactive, Thermo Scientific; Angelidis et al., 2019). In these analyses, approximately 2 μg of peptides were separated in a 4 h gradient on a 50 cm long (75 μm inner diameter) column packed in‐house. Further details of the LC–MS/MS analyses performed on these aged mice samples are detailed in Angelidis et al. (2019). For the purposes of this work, 3‐month‐old mice were considered the young control group, while mice aged 22 and 24 months were considered the old mice group. Similarly, data from tissue fractionations originally performed in the study of (Angelidis et al., 2019) were combined and analyzed to allow the bioinformatics profiling of whole tissue proteomes.

#### 2.4.2 Mass spectrometry data analysis

Analysis of proteomics raw data for the identification of t‐Cys in control and aged mice LC–MS/MS proteomes was carried out using in‐house Mascot server (version 2.6.02, Matrix Science, MA). Database search in Mascot was performed allowing a precursor tolerance of 10 ppm and fragment ion tolerance of 30 ppm. Trioxidized Cys, deamidation of asparagine (Asp) and glutamine (Gln), oxidation of metionine (Met), and carbamidomethylation of Cys were set as variable modifications. Data were searched against the UniProt mouse database (91,089 sequences and 38,788,886 residues). Analysis of proteomics raw data for the identification of p‐Ser was performed using PEAKS Studio X Pro software (version 10.6 Bioinformatics Solutions). PEAKS PTM algorithm available in PEAKS Studio software was used for the identification of protein PTMs. FDR <1% was established for protein identification in all samples. Carbamidomethylation of Cys was set as fixed modifications. A precursor tolerance of 10 ppm and fragment ion tolerance of 0.05 Da was allowed in the searches. Data were searched against the UniProt mouse database (55,466 sequences downloaded in Nov 2020). Only peptides containing p‐Ser modified residues with AS score of 1000, indicating maximum identification confidence in PEAKS algorithm, were considered (Han et al., 2011). Database search results obtained were thoroughly exported into comma separated values files for further analysis.

The analysis of the data was performed using R software (version 4.2.1). The list of R packages used in this study is included in Table S1. Protein structures were downloaded from the Protein Data Bank (PDB) when available, or from the Alphafold database. The homogeneity of the obtained data from the analyzed mice proteomes was assessed using the Brown–Forsythe test. In cases where non‐parametric analysis was necessary, one‐way ANOVA on ranks was performed, with statistical significance set at *p* < 0.05, unless otherwise indicated. Data were further analyzed independently using parametric one‐way ANOVA with Tukey's test for multiple comparisons. Statistical significance was set at corrected *p* < 0.05, unless stated otherwise. Proteins that exhibited significant differences, based on spectral count of t‐Cys and the aforementioned statistical criteria, between young and old groups, were further assessed by advanced bioinformatics (Robinson et al., 2010). Samples from male and female animals were collectively analyzed unless otherwise specified in specific analyses aimed to scrutinize the effect of biological sex.

#### 2.4.3 Predictive kinase interactions and molecular dynamics simulations

To identify the predicted enzyme interactions with significantly modulated p‐Ser and t‐Cys residues in the aged proteomes, we used GPS 5.0 (Wang et al., 2020). Simulations were performed by using 21‐amino acid sequence fragments containing the specific p‐Ser or t‐Cys site ±10 amino acids. Generation of fragments was done in Pymol (version 2.5.4). t‐Cys residues, within the analyzed sequences, were subject to amino acid substitution by Ser. The high threshold option was selected in GPS software for each prediction. Only the three enzymes with higher predictive scores for each modified site were considered in these analyses.

For molecular dynamics (MD) simulations, the relevant protein structures were downloaded from PDB, when available, or alternatively from Alphafold database. Vienna PTM 2.0 (Margreitter et al., 2013) was used to introduce the PTMs object of study into PDB protein structures and cysteic acid was used to simulate t‐Cys residues. Protein docking was performed in Hdock webtool server (Yan et al., 2020) using the sequence fragments containing the modified site ±10 amino acids extracted from the post‐translationally modified PDB protein structures. MD simulations were carried out using GROMACS software (version 2021.4‐Ubuntu‐2021.4‐2; Van Der Spoel et al., 2005), as described (Margreitter et al., 2017), adapted to t‐Cys and p‐Ser. Briefly, water molecules were firstly removed from PDB protein structures. The GROMOS‐ffG54a8 force field was used. MD simulations were performed in a cubic box model of water, in which Na‐ and Cl‐ ions were added to neutralize the charges of the protein fragment residues. For MD simulations, the initial system energy minimization and temperature equilibrium steps were performed with a 100 ps run under constant number of particles, volume, and temperature (NVT) conditions. Subsequently, another 100 ps run under constant number of particles, pressure, and temperature (NPT) conditions using the Parrinello‐Rahman barostat was performed. The final run was carried out with 1 ns. After the trajectory correction, the root‐mean‐square deviation of atomic positions (RSMD) was adjusted to protein backbone.

### 2.5 Cell culture and neutral red uptake assay

HEK293 (ATCC CRL‐1573™) and SHSY5Y (ATCC CRL‐2266™) were grown using T75 flasks in growth medium (GM) [DMEM high glucose supplemented with 10% of fetal bovine serum (FBS) and 1% of penicillin/streptomycin (PS)], until reaching 80–90% confluence. For the oxidative stress experiments, cells were cultured in 6‐well plates (20,000 cells per well) for 24 h to reach confluence. GM was changed and supplemented with 250 or 500 μM of hydrogen peroxide (H_2_O_2_) for 24 h. Then, cells were washed twice with cold 1× PBS. To assess cellular activity and viability, we performed a neutral red uptake assay as previously indicated without modifications (Repetto et al., 2008).

### 2.6 Bicinchoninic acid protein determination assay

Culture cells washed with cold PBS were lysed using 0.1% Triton X‐10 and protease inhibitors. Proteins were extracted using centrifugation at 12,000 × *g* for 10 min by taking the supernatant. To analyze the amount of protein in the cell lysis supernatants or skin tissue homogenates, BCA protein assay was performed, according to the manufacturer's instructions. Briefly, 25 μL of each sample was loaded in a 96‐well plate, followed by 200 μL of BCA/copper complex solution. The absorbance was measured at 562 nm in a microplate reader.

### 2.7 IKKα (CHUK) kinase activity assay

For the kinase activity measurement, 100 μg of protein obtained from cell lysates and 4 ng of IKKα were submitted to the IKKα kinase assay (V4068, Promega Biotech Ibérica S.L.) following the manufacturer's instructions. Results of kinase activity were represented by the % of produced ADP. This was measured using ADP‐Glo™ Kinase Assay (V6930, Promega Biotech Ibérica S.L.) following the manufacturer's instructions.

## 3 RESULTS

### 3.1 Aging induces the accumulation of t‐Cys in mammalian proteomes

**FIGURE 1:**
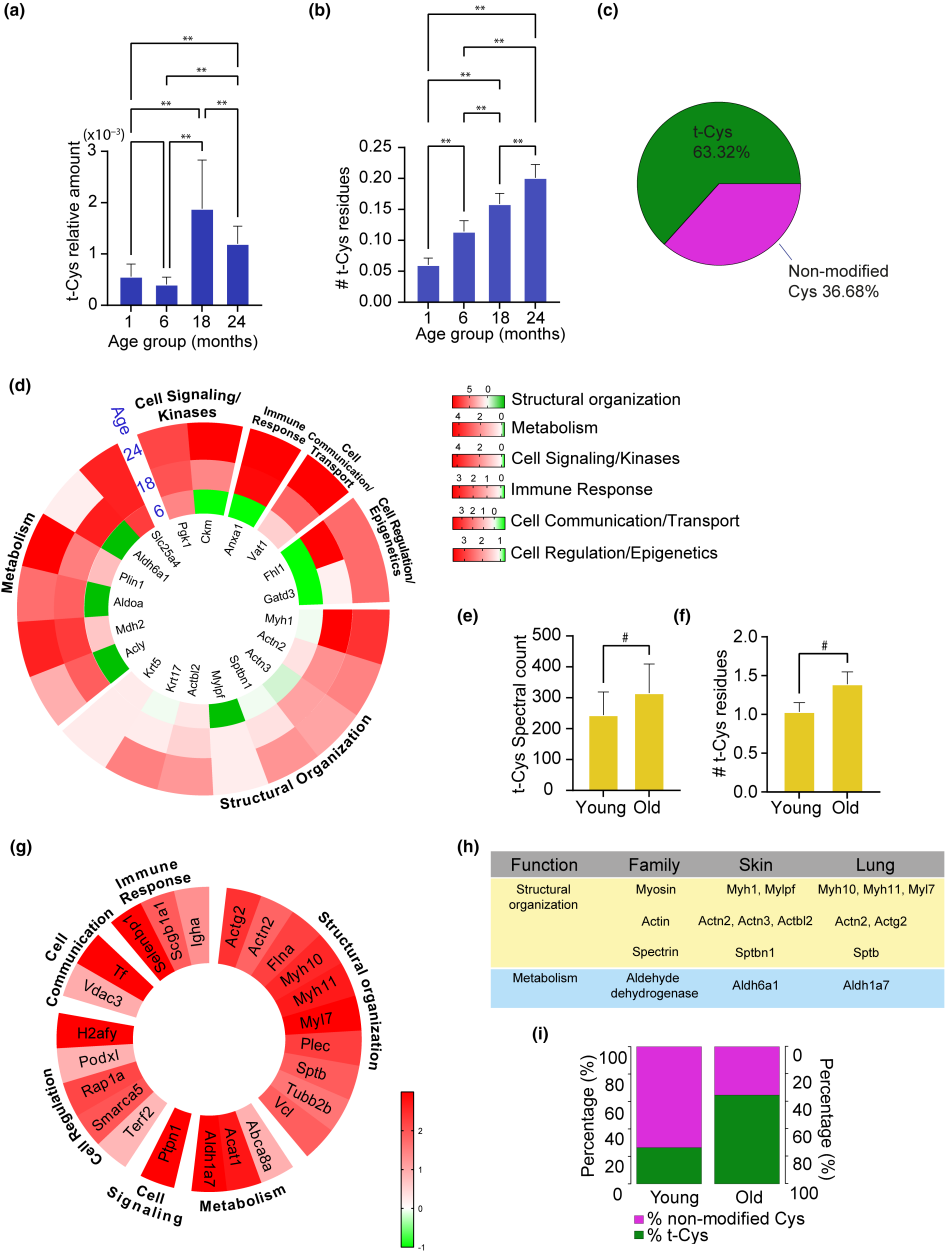
In‐depth proteomic characterization and functional categorization of t‐Cys affected proteomes in murine aging skin and lung. (a). Normalized average level distributions of t‐Cys residues in the skin proteomes measured by spectral count. (b) Normalized average count of t‐Cys residues in the skin proteomes. (c) Pie chart representing the averaged stoichiometry of the t‐Cys containing sites relative to unmodified Cys from the total proteome. (d) Concentric heatmap indicating the proteins that contain significant differentially expressed t‐Cys residues in the murine skin proteome aging groups compared with controls (1 month). Protein gene symbols are indicated in the inner part of the heatmap and their cellular function in the outer part of the heatmap. (e) Average level distributions of t‐Cys residues in the lung proteomes measured by spectral count. (f) Average count of t‐Cys residues in the analyzed murine lung proteomes. (g) Circular heatmap indicating the proteins that contain differentially expressed t‐Cys residues and their cellular function (outer part of the heatmap) in the murine lung proteome of the aged group compared with young animals. (h) Comparative analysis between trioxidized age‐dependent significantly modulated proteins identified in skin and lung. Protein isoforms were not taken into account to highlight the common gene families between both sets of data. (i) Averaged stoichiometry of the t‐Cys containing sites relative to unmodified Cys in trioxidized age‐dependent modulated proteins. *Indicates parametric significant differences at *p* ≤ 0.05; **Indicates parametric significant differences at *p* ≤ 0.001; ^#^Indicates non‐parametric significant differences at *p* ≤ 0.05. Error bars in graphs represent the standard error of the mean (SEM).

We initially screened the skin proteomes of aging mice to investigate a potential age‐dependent variation in the cumulative levels of t‐Cys and the total number of t‐Cys residues. As showed in Figure 1a, we observed a significant increase in cumulative t‐Cys levels in the proteomes of aging and aged mice compared with the young groups. Similarly, Figure 1b demonstrated a significant increase in the total number of t‐Cys residues per protein in the proteomes of aging and aged mice. The stoichiometry of t‐Cys sites relative to unmodified Cys at the proteome‐wide level was also analyzed, revealing that the modified proteins were, on average, modified by 63.32% of their total (Figure 1c; Table S2). We also found that biological sex significantly modulates the total number of t‐Cys residues in the analyzed proteomes (Table S3). Our focus then shifted to analyzing what kind of proteins were affected by t‐Cys (Figure 1d). Functional categorization revealed that these proteins play important roles in multiple cellular functions, including: glycolysis (fructose‐bisphosphate A (ALDOA), metabolic regulation‐related proteins (ATP‐citrate synthase (Acly) and malate dehydrogenase 2 (Mdh2), cell structure (keratin, type II cytoskeletal 5 (Krt5), alpha‐actinin‐2 (Actn2), and myosin 1 (MYH1), and immunity (immunoglobulin heavy constant alpha (IGhA) and annexin A1 (ANXA1). Moreover, we found that dysregulated t‐Cys residues also impact on cell signaling kinases, such as creatine kinase M‐Type (CKM) and phosphoglycerate kinase 1 (PGK1; Figure 1d).

To substantiate these results, we performed further validation experiments using an alternative cohort of aging mice. This validation experiments confirmed that aging does indeed modulate the occurrence of t‐Cys in the mice proteomes, as showed in Figure 1e,f. Moreover, our investigation revealed that the presence of t‐Cys at the protein level during aging is associated with the affectation of similar molecular functions across diverse proteomes, including cell signaling, immune response, and cell metabolism (Figure 1g). Specifically, we found that the same families of enzymes were affected by aging‐dependent t‐Cys across the various analyzed proteomes and mice cohorts, including several Myosin isoforms and related Myosin regulation proteins (Myh1, Myh10, Myh11, Mylpf, and Myl7), Actins (Actn2, Actn3, Actbl2, and Actg2), Spectrins (Sptbn1 and Sptb), and Aldehyde dehydrogenases (Aldh6a1 and Aldh1a7; Figure 1h). Additional details of the proteins displaying significant age‐dependent modulation in their t‐Cys profiles are included in Tables S4 and S5. The stoichiometry of t‐Cys sites relative to unmodified Cys in these trioxidized age‐dependent modulated proteins was also calculated. Although in the young groups (1 and 6 months), the percentage of the modified fraction of the protein was 24.44%, this stoichiometry increased to 64.74% of the total protein modified in the old groups (18 and 24 months; Figure 1i; Table S6).

### 3.2 Aging modulates p‐Ser and its interaction with t‐Cys

**FIGURE 2:**
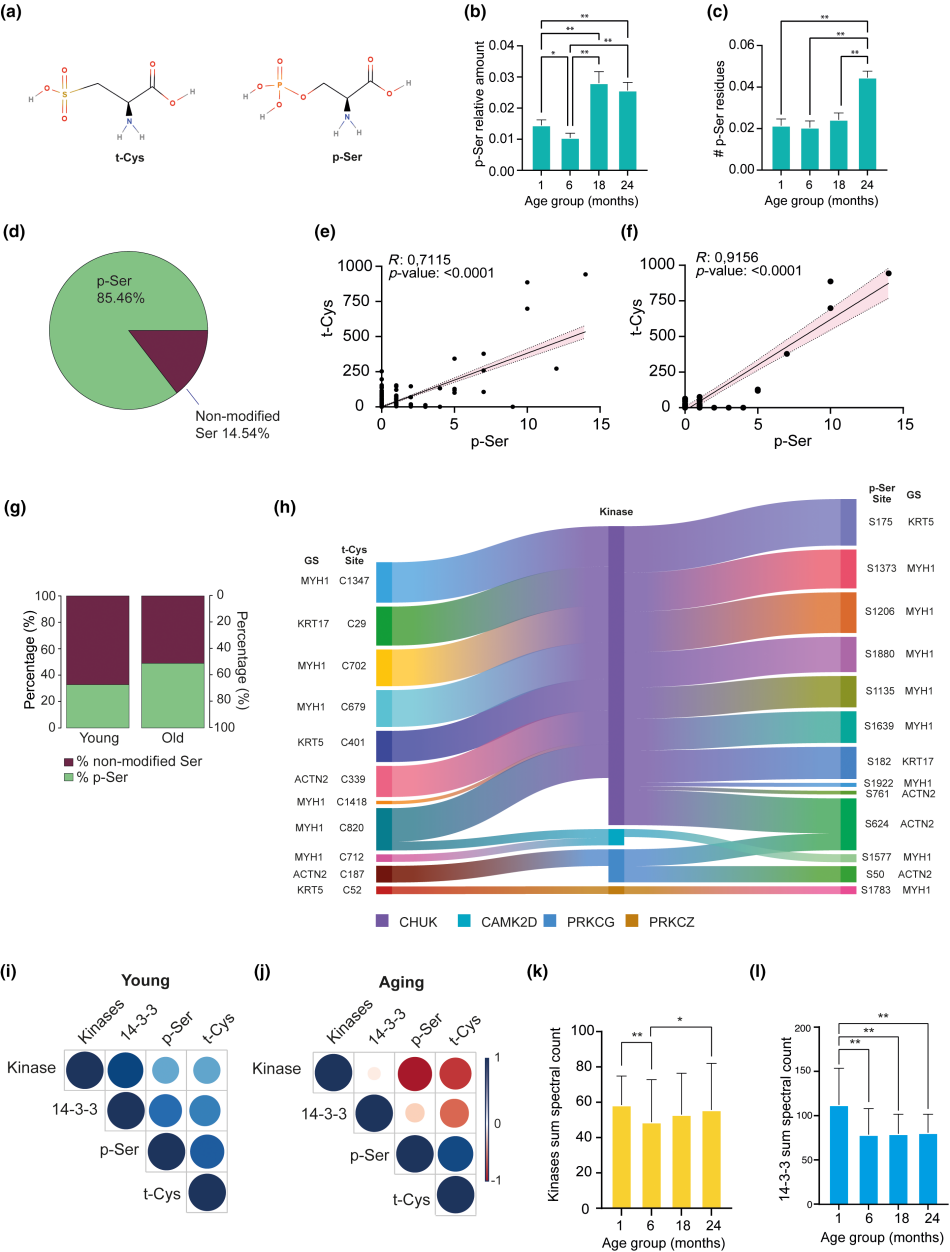
Characterization of the cross‐effects and functional interplays occurring between t‐Cys and p‐Ser in the aged murine proteome. (a) Molecular structure of t‐Cys and p‐Ser illustrating the high molecular similarities established between both post‐translationally modified residues. (b) Normalized average level distributions of p‐Ser residues in murine proteomes measured by spectral count. (c) Normalized average count of p‐Ser residues in murine proteomes. (d) Pie chart representing the averaged stoichiometry of the P‐Ser containing sites relative to unmodified Ser from the total proteome. (e) Correlation graph demonstrating positive strong association between t‐Cys and p‐Ser levels at proteome‐wide in aged animals. (f) Correlation graph demonstrating near‐complete positive association between the t‐Cys and p‐Ser levels, considering only those proteins that showed significantly altered levels (*p* < 0.05) of t‐Cys and p‐Ser in the proteome of aged animals. (g) Sankey diagram showing the interaction between kinases (center) and t‐Cys containing proteins (left) or p‐Ser containing proteins (right). Gene symbol and modified site location are indicated for t‐Cys and p‐ser affected proteins. Only the proteins found in the aged murine proteomes with significantly increased t‐Cys and p‐Ser levels compared with controls are included. Only kinases with the highest score chance of interaction in GPS 5.0 software with both analyzed t‐Cys and p‐Ser sites were included. Stripe thickness in graph represents relative levels of predicted interaction of each respective kinase with each respective t‐Cys and p‐Ser residue. Correlation matrices depicting the associations of p‐Ser and t‐Cys with the protein level of predicted kinases and 14‐3‐3 proteins in (h) young murine proteomes (1–6 months) compared with (i) aging murine proteomes (18–24 months). The color and thickness of the matrix dots represent Pearson's *r* value. Imbalance between the levels of the analyzed kinases and 14‐3‐3 proteins and the ability of interact with t‐Cys and p‐Ser sites in the proteome of young mice compared with aging mice was demonstrated by the respective positive and negative matrix dots observed. (j) Proteome levels distribution of the t‐Cys and p‐Ser interacting kinase enzymes identified in the murine proteomes and with interacting ability predicted by GPS 5.0. (k) Proteome levels of the p‐Ser interacting 14‐3‐3 proteins identified in the analyzed murine proteomes. (l) Averaged stoichiometry of the p‐Ser containing sites relative to unmodified Ser in trioxidized age‐dependent modulated proteins. *Indicates parametric significant differences at *p* ≤ 0.05; **Indicates parametric significant differences at *p* ≤ 0.001. Error bars in graphs represent SEM.

As t‐Cys residues closely resemble p‐Ser sites in their molecular structures, as depicted in Figure 2a, we conducted a thorough examination to identify any age‐dependent occurrence of p‐Ser in the proteomes of aging mice. Indeed, in line with our previous observations of t‐Cys, we discovered a significant age‐dependent increase affecting both the cumulative level and total number of p‐Ser residues in the analyzed proteomes (Figure 2b,c; Table S7). The stoichiometry of p‐Ser sites relative to unmodified Ser at the proteome‐wide level was also analyzed, revealing that the modified proteins were, on average, modified by 85.46% of their total (Figure 2d; Table S8). To gain deeper insight into the potential implications of these findings, we conducted an analysis to determine whether a significant association could be established between the aging proteomes involving t‐Cys and p‐Ser. Our results revealed a strong and significant positive correlation between the levels of both PTMs at the proteome‐wide level (Pearson's *r* = 0.71, *p* < 0.0001; Figure 2e). Furthermore, the correlation effect between t‐Cys and p‐Ser was almost complete when considering only the proteins that exhibited significant modulation in their levels of t‐Cys from the analyzed proteomes (Figure 2f). The stoichiometry of p‐Ser sites relative to unmodified Ser in the proteins showing age‐dependent modulation in phosphorylation was also calculated. Although in the young groups (1 and 6 months), the percentage of the modified fraction of the protein was 39.79%, and this stoichiometry increased to 60.33% of the total protein modified in the old groups (18 and 24 months; Figure 2g; Table S9). We also observed that, although certain t‐Cys and p‐Ser residues may be distant from each other in the primary structure of the protein, they are not far apart in the tertiary structure of the molecule. A clear example of this fact includes the residues t‐Cys 187 and p‐Ser 50 in ACTN2, which are separated by 137 residues in the primary structure but only 20.9 Å apart in the tertiary structure (further details provided in Figure S1).

### 3.3 p‐Ser‐related enzymes interact with t‐Cys residues in the aging proteome

Having established the close biochemical resemblance and significant association between t‐Cys and p‐Ser residues in the aging proteome, our next step was to analyze which p‐Ser‐related enzymes displayed a potential ability to interact with t‐Cys. For this analysis, we focused specifically on proteins that contained both t‐Cys and p‐Ser residues from the identified age‐dependent significantly modulated proteins, as outlined in detail in Table 1. We found that the enzymes conserved helix–loop–helix ubiquitous kinase (CHUK), calcium/calmodulin dependent protein kinase II delta (CAMK2D), protein kinase C gamma (PRKCG), and protein kinase C zeta (PRKCZ) exhibited a higher likelihood of interacting with both t‐Cys and p‐Ser residues in the aging proteome, as depicted in Figure 2h.

**TABLE 1:** Descriptive analysis of differentially expressed proteins containing both t‐Cys and p‐Ser modifications.

GS	Protein description	t‐Cys spectral count						p‐Ser spectral count					
		1	6	18	24	*F*	*p*‐value	1	6	18	24	*F*	*p*‐value
Myh1	Myosin‐1	–	–	842.67 ± 127.64	208.33 ± 147	199.58	1.55E‐10	1.67 ± 0.58	3 ± 1	11.33 ± 2.3	5.67 ± 1.54	32.4	1.91E‐05
Krt5	Keratin, type II cytoskeletal 5	25 ± 0	47.67 ± 6.35	75.67 ± 1.53	59 ± 8.66	3.49	4.98E‐02	–	–	1 ± 0	0.33 ± 0.58	16.24	3.68E‐04
Krt17	Keratin, type I cytoskeletal 17	–	–	5.67 ± 9.81	22 ± 1	24.15	2.20E‐05	–	0.67 ± 0.58	1 ± 0	–	12.06	1.19E‐03
Actn2	Alpha‐actinin‐2	–	2.67 ± 4.71	17.33 ± 2.87	21 ± 4.36	21.02	4.44E‐05	–	–	1 ± 0	–	110.38	6.21E‐08

*Note*: For every protein in every age group (1, 6, 18, 24 months) the spectral count mean ± SD for t‐Cys (left) and p‐Ser (right) are indicated. ‐ represent non detected values. Significant differences were assessed by ANOVA (*p* < 0.05). The Fisher (*F*) and *p*‐value for the differential expression analysis are detailed for the t‐Cys and p‐Ser spectral count comparisons.

Furthermore, through a careful examination of the association effect between the levels of p‐Ser‐related enzymes, t‐Cys, and p‐Ser residues, we uncovered a reversed pattern of correlation between kinases and the target post‐translationally modified residues between the young and old groups (Figure 2i,j). Intriguingly, the 14‐3‐3 family of p‐Ser‐related enzymes exhibited a kinase‐like pattern of correlation with the investigated post‐translationally modified residues in the young and old groups, as depicted in Figure 2i,j. Although no clear differences were observed in terms of the cumulative protein levels of the aforementioned p‐Ser‐related enzymes identified in this study (Figure 2k,l), a relatively higher level of these proteins in the youngest group could tentatively be established (Figure 2k,l).

### 3.4 t‐Cys mimic p‐Ser to influence protein molecular dynamics in aging

**FIGURE 3:**
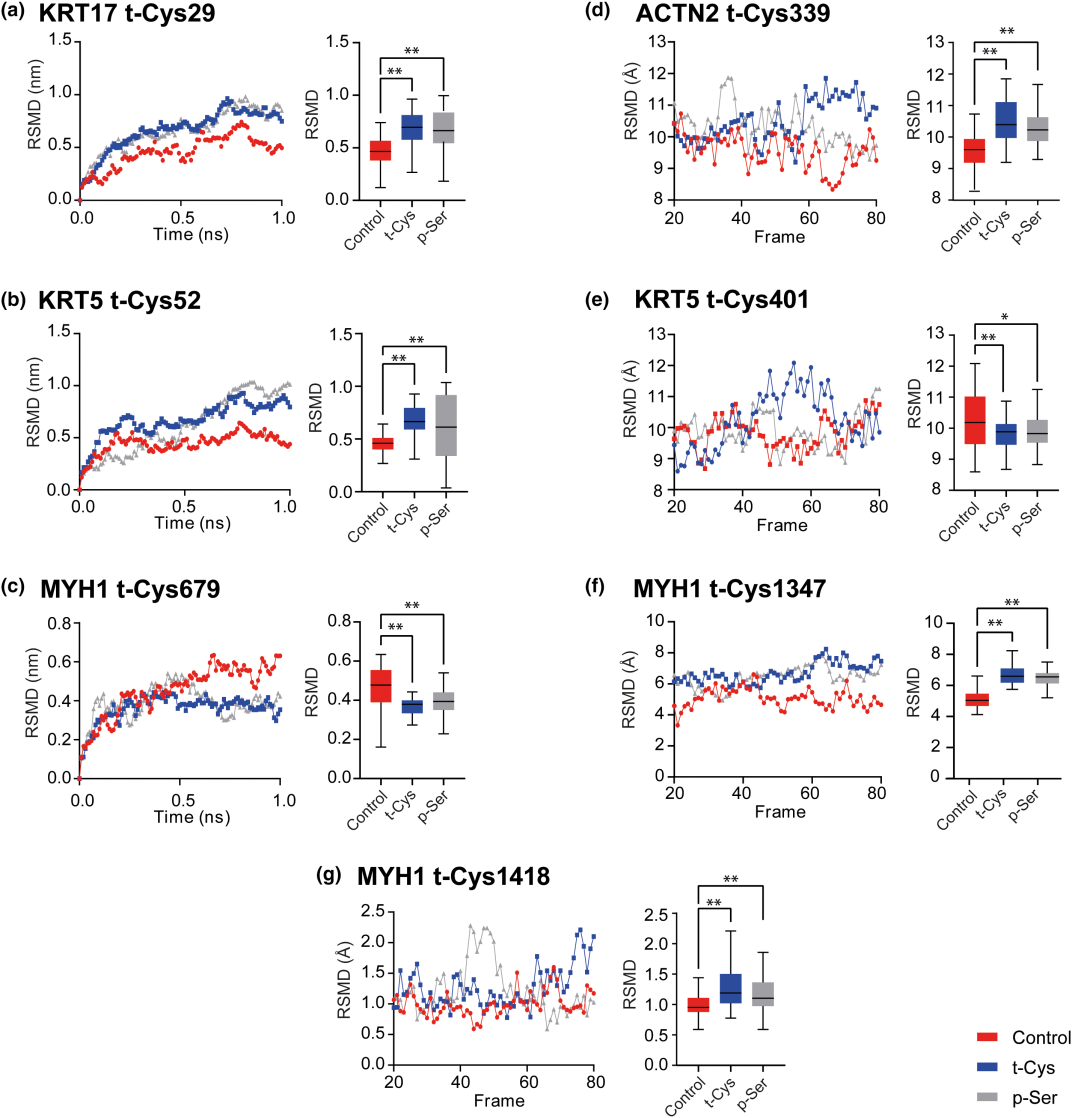
Simulated structural dynamics of the proteins with significantly increased levels (*p* < 0.05) of t‐Cys in murine aging proteomes. Root‐mean‐square deviation (RSMD) of (a) KRT17 (t‐Cys29), (b) KRT5 (t‐Cys52) and (c) MYH1 (t‐Cys679) were calculated using GROMACS software, and RMSD of proteins (d) ACTN2 (t‐Cys339), (e) KRT5 (t‐Cys401), (f) MYH1 (t‐Cys1347) and (g) MYH1 (t‐Cys1418) were calculated using VMD Trajectory tool. For every protein the RMSD simulations were performed with (i) the protein containing the t‐Cys residues (blue), (ii) the protein with t‐Cys residue substituted by a p‐Ser residue (grey) and (iii) the unmodified protein (Control – red). The protein structure including the identified t‐Cys residues ±10 amino acids was used in all the performed simulations. All simulations were performed under constant number of particles, volume, temperature and pressure conditions. *Indicates significant RMSD parametric statistical differences at *p* ≤ 0.05; **Indicates significant RMSD parametric statistical differences at *p* ≤ 0.001.

To investigate whether t‐Cys, which were found to be significantly modulated by aging in this study, have a comparable effect on protein structure to that of p‐Ser, we have performed in silico molecular dynamic simulations. In these experiments, we evaluated the protein structure similarity using the root‐mean‐square deviation (RMSD) measurement, that is the most common quantitative measure of the similarity between two superimposed atomic coordinates (Kufareva & Abagyan, 2012). Importantly, our results showed no significant changes in the effects of t‐Cys and p‐Ser on the structure of the simulated proteins, as depicted in Figure 3a–g, indicating t‐Cys can mimic the protein structural effects of p‐Ser. Therefore, we developed a function to calculate a t‐Cys/p‐Ser similarity index (TPSi), thoroughly defined in Figure S2. Based on these calculations, TPSi for all the significantly age‐related modulated t‐Cys residues in the analyzed proteomes was higher than 0.8 (Table S10).

### 3.5 t‐Cys shows ability to interfere p‐Ser‐mediated cellular signaling

**FIGURE 4:**
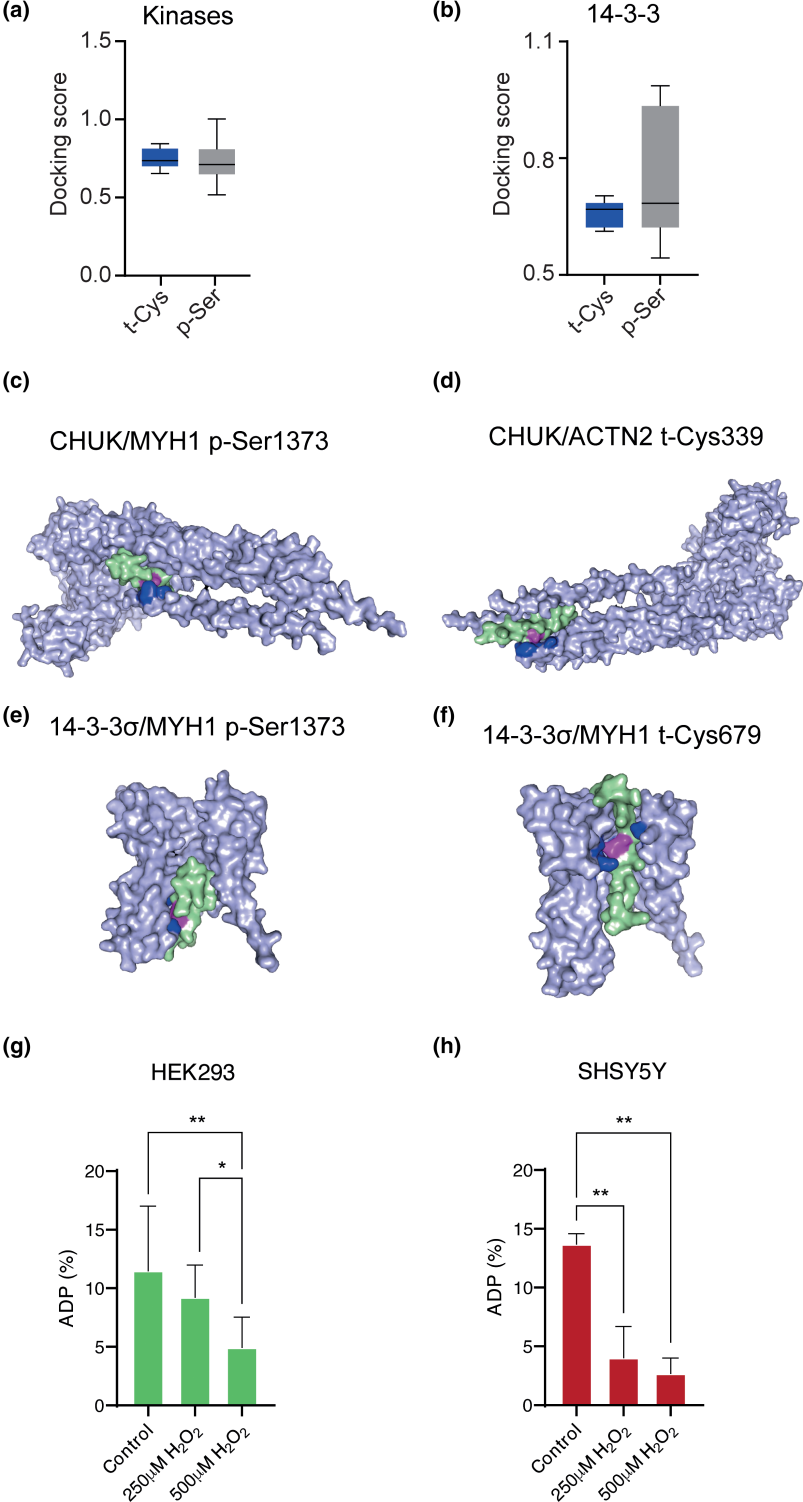
Protein–protein docking simulations of p‐Ser‐interacting enzymes (kinases and 14‐3‐3 proteins predicted using GPS software) with t‐Cys containing proteins that were identified significantly increased in the profiled aging murine proteomes. (a) Graph indicating the mean docking score values of the simulated interaction between predicted kinases with the identified t‐Cys and p‐Ser protein regions. (b) Graph indicating the mean docking score values of the simulated interaction between 14‐3‐3 σ protein with the identified t‐Cys and p‐Ser protein regions. Representative kinase docking model with the highest docking score involving the protein–protein interaction of (c) the conserved helix–loop–helix ubiquitous kinase (CHUK; grey) with the protein region of the myosin heavy chain 1 (MYH1; green) containing the t‐Ser1373 (magenta). The amino acids in CHUK kinase that participates in the interaction with t‐Ser1373 of MYH1 are displayed in blue. (d) the CHUK kinase (grey) with the t‐Cys339 (magenta) of the conserved actinin 2 protein (ACTN2; green). The amino acids in CHUK kinase that participates in the interaction with t‐Cys339 of ACTN2 are displayed in blue. (e) the 14‐3‐3 σ protein (grey) with the p‐Ser1373 (magenta) of the MYH1 (green). The amino acids in 14‐3‐3 σ protein that participates in the interaction with p‐Ser1373 of MYH1 are displayed in blue. (f) the 14‐3‐3 σ protein (grey) with the t‐Cys residue 679 (magenta) of the MYH1. The amino acids in 14‐3‐3 σ protein that participates in the interaction with MYH1 are displayed in blue. Significance was established at *p* < 0.05 and no significant differences were observed on the docking scores obtained involving kinases and 14‐3‐3 σ protein with the murine aging proteome differentially modulated p‐Ser and t‐Cys residues. The protein structure including the identified t‐Cys or p‐Ser residues ±10 amino acids was used in all the performed docking simulations. In vivo evaluation of the CHUK kinase activity in cumulative oxidized proteomes assessed for (g) HEK293 cells and (h) SHSY5Y cells. Oxidative stress was generated by treating cells with 250 μM H_2_O_2_ and 500 μM H_2_O_2_ compared with untreated cells (Control). CHUK kinase activity was assessed by IKKα (CHUK) kinase activity assay.

Finally, to ascertain whether the previously predicted p‐Ser‐related enzymes (Table S11), including kinases and 14‐3‐3 proteins, can interact with aging‐modulated t‐Cys in the mammalian proteome, we conducted in silico molecular dockings, an excellent approach used to model the interaction between molecules at atomic level (Meng et al., 2011). Strikingly, these experiments confirmed that t‐Cys possesses the same ability as p‐Ser to interact with kinases (Figure 4a; Table S12). Notably, competitive interaction has been identified when both post‐translationally modified residues are closely located within the primary structure of the protein (Table S13). Furthermore, the capacity of interaction with t‐Cys residues was also extrapolated to 14‐3‐3 proteins (Figure 4b; Table 2). The molecular dockings of the representative predicted kinases and 14‐3‐3 proteins with the identified aging‐modulated t‐Cys residues are depicted in Figure 4c–f, respectively, and in Figures S3 and S4.

**TABLE 2:** Docking scores of the interaction between 14‐3‐3 σ protein and t‐Cys or p‐Ser containing protein regions.

GS	Protein description	Site docking score					Site docking score			*p*‐value
		t‐Cys					p‐ser			
Myh1	Myosin‐1	1347 t*‐0.612 ± 0.032	679*t‐0.669 ± 0.056	702 t*‐0.619 ± 0.010	712 t*‐0.685 ± 0.024	820 t*‐0.704 ± 0.016	1373p*‐0.986 ± 0.003	1639p*‐0.622 ± 0.063	1922p*‐0.934 ± 0.021	0.0705
Krt5	Keratin, type II cytoskeletal 5	52*t‐0.684 ± 0.050	–	–	–	–	175p*‐0.665 ± 0.063	–	–	0.9097
Krt17	Keratin, type I cytoskeletal 17	29 t*‐0.684 ± 0.050	–	–	–	–	182p*‐0.544 ± 0.072	–	–	0.0565
Actn2	Alpha‐actinin‐2	187 t*‐0.641 ± 0.029	339 t*‐0.625 ± 0.061	–	–	–	50p*‐0.684 ± 0.050	761p*‐0.797 ± 0.048	–	0.1991

*Note*: Docking scores were obtained uploading to Hdock server PDB structures of the fragments of the proteins with t‐Cys or p‐Ser modifications and 14‐3‐3σ. It is shown the proteins gene symbol (GS), protein description, Mean ± SD of the PTM Site Docking Score of the three best models and P‐value. Significance was assessed by unpaired t test, with a minimum significance level *p* < 0.05. *t = t‐Cys, p = p‐Ser.

After the in silico observations detailed above, we then conducted in vitro assays to identify any oxidation‐dependent modulation of the CHUK kinase activity. As shown in Figure 4g,h, significant oxidation‐dependent decay in CHUK kinase activity was consistently found in all analyzed human cell line proteomes. As expected, and thoroughly detailed throughout this work, this test tube generated result underscores the high dependence of CHUK kinase activity on the interplay within the cellular proteome, involving oxidative stress and the requirement for homeostatic phosphorylation signaling. Additionally, cellular viability tests involving the analyzed human cell lines proteomes were also performed and oxidative stress dependent cell death, as expected, was clearly identified (Figure S5).

Finally, we also investigated which specific residues in the primary structure of the kinases more commonly interact with t‐Cys and p‐Ser residues, respectively. Strikingly, we found that the residues interacting with both post‐translationally modified residues were the same, although at different proportions, as follows: t‐Cys sites: Leucine (12.17%), Glutamic acid (8.98%), and Arginine (8.95%); p‐Ser sites: Arginine (16%), Glutamic acid (11.67%), and Leucine (10.67%; Table 3). Similarly, we analyzed the interacting residues in the enzyme proteins and the proteins containing significantly aging‐modulated t‐Cys residues to determine whether these belong to any functional domains within the tertiary structure of the proteins. These analyses revealed that several of the interacting sites are in structurally functional domains, including relevant enzyme and peptide binding sites as detailed in (Tables S14 and S15).

**TABLE 3:** Amino acid interaction preferences for t‐Cys and p‐Ser sites.

Residue	Interaction (%)	
	p‐Ser	t‐Cys
Arg	16	8.98
Glu	11.67	8.98
Leu	10.67	12.17
Ile	6.67	5.5
Gln	6.67	5.79

*Note*: The averaged percentage of the top five interacting residues were obtained from the docking simulations performed between every t‐Cys or p‐Ser sites identified in the age‐dependent modulated proteins and all predicted kinases and 14‐3‐3σ protein. Docking simulations were performed using the protein structure including the identified t‐Cys or p‐Ser residues ±10 amino acids. The interaction preference is expressed as percentage of interaction of a specific amino acid over the total amino acids that interacted with the modified residues calculated from all docking simulations.

## 4 DISCUSSION

Aging plays a crucial role in the development of several major human diseases, such as neurodegeneration, cardiovascular disease, and cancer (Gallart‐Palau et al., 2019; Li et al., 2021; Lorca, Laparra, et al., 2022; Lorca, Mulet, et al., 2022; Park et al., 2019). At the molecular level, dysregulated phosphorylation‐mediated abnormal cellular signaling has been extensively associated with the influence of aging on these diseases (Ardito et al., 2017; Bilbrough et al., 2022; Gallart‐Palau et al., 2019). Similarly, increased oxidative stress is widely recognized to relate to aging and the onset and progression of these human disorders (Ebert et al., 2022; Liguori et al., 2018; Roy et al., 2023). However, the underlying molecular mechanism remains elusive. This study establishes, for the first time, a previously unconceived molecular‐level connection between oxidative stress generated t‐Cys and p‐Ser as two interfering fundamental mechanisms in age‐related pathology. This connection was supported by novel proteomic results from aging tissues, initially confirmed by their almost identical biochemical profile and further revealed through state‐of‐the‐art in silico experiments. As a result, t‐Cys emerges as a potential disruptor of the delicate, age‐related homeostatic cell signaling mediated by p‐Ser. That uncovering carries significant implications for the onset and progression of several major aging‐related diseases, which will be further discussed in this context. Moreover, these compelling findings highlight the vital roles of t‐Cys in human diseases by actively interfering with cellular signaling mediated by phosphorylation, rather than simply causing a loss of function to the affected proteins as currently thought. Consequently, we believe that these findings pave the way for further research in several human diseases where p‐Ser and exacerbated oxidative stress could have been implicated. Moreover, the findings presented in this study broaden our understanding of redox signaling in the context of aging, shedding light on an essential aspect with significant implications for comprehending the molecular foundations of various age‐related diseases (Lennicke & Cochemé, 2021; Wall et al., 2012; Zuo et al., 2022).

Our experimental data confirmed that t‐Cys levels increase in the proteome of skin cells during the aging process. This result is consistent with our previously reported in brain tissues proteomes (Guo et al., 2020), and by other colleagues in other proteomes (Angelidis et al., 2019; Garner & Spector, 1980; Pérez, Buffenstein, et al., 2009). This finding was also confirmed in this study by re‐analysis of publicly available proteomics data of aging murine origin, confirming its consistency in several mice proteomes as consequence of exacerbated oxidative stress (Angelidis et al., 2019). Of note, Pérez et al. in line with our findings, additionally found that total Cys available is diminished with age due to irreversible oxidation (Pérez, Buffenstein, et al., 2009). This has significant implications for the delicate balance of cellular protein synthesis required in aging and several pathological processes (Rizvi & Maurya, 2008). Similarly, in this study, we observed that t‐Cys directly dysregulates proteins implicated in cell metabolism, cell structure, and systemic immunity in the skin proteome in an age‐dependent manner. These findings may also provide an explanation for the aging‐related dysregulation of skin proteins implicated in the aforementioned cellular and systemic functional domains previously reported (Ma et al., 2020). Similarly, although other colleagues have found that cysteic acid may not be modulated by aging in certain organs (Benedetti et al., 1991), here we have found that t‐Cys has highly similar stoichiometric parameters to phosphorylation. This similarity suggests that t‐Cys, like phosphorylation, becomes influenced by aging and may have relevant biological significance for the proteins affected, reaching over 60% of the total identified modified protein in average.

Indeed, our data initially suggested that t‐Cys may function as a potential interfering mechanism in aging‐related p‐Ser mediated cell signaling. This was supported by the consistent increase in the occurrence of p‐Ser, along with the age‐related rise in t‐Cys, within the profiled murine skin proteomes. Additionally, we also observed significant and robust associations between the levels of these two DPMs across the analyzed proteomes. These remarkable findings were also here accompanied by an abnormal pattern of association of these modified residues with the levels of the p‐Ser‐related enzymes protein kinases and 14‐3‐3 proteins. While positive correlations between the mentioned PTMs and p‐Ser‐related enzymes were observed in the young groups, this pattern reversed to negative correlations in the analyzed aged groups. These findings raise the hypothesis that these enzymes may be insufficient to facilitate the required interactions for balanced cell signaling with p‐Ser during aging. Furthermore, the increase in t‐Cys sites, which, based on our in silico experiments, has the ability to interact with the p‐Ser‐related enzymes just like p‐Ser does, may contribute to this phenomenon. Further research is necessary to investigate this central hypothesis that has emerged from the development of this work. Additionally, the data generated from these in silico experiments indicate the existence of competitive kinase binding when both residues are closely located in the primary structure of the protein (within a distance of <25 residues). Notably, the three most common residues in the kinase that interact with both t‐Cys and p‐Ser include Arginine, which carries a positive charge, observation that seems to imply the involvement of the negative charge of the post‐translationally modified residues in the interaction.

In a related context, one of the p‐Ser‐related enzymes identified in this study, which has been robustly proven in silico to interact with both t‐Cys and p‐Ser, is the kinase CAMK2D. The role(s) of CAMK2D in aging are not well understood, but it has been reported that during aging, the activity of this kinase increases in cardiac tissue, along with elevated pro‐inflammatory markers and oxidative stress (McCluskey et al., 2019). These findings indicate that the alterations in the signaling pathways of the kinase identified in this study directly contribute to the dysregulation of proteome signaling observed in certain proteomes associated with the aging process. Another crucial group of proteins that engage with phosphorylation sites are the 14‐3‐3 proteins. These proteins recognize and interact with specific phosphorylated protein sites, actively participating in the regulation of various biological processes, including apoptosis, cell cycle progression, proliferation, transcription, DNA replication, ion channel function, and cytoskeletal organization (Fan et al., 2019; Fu et al., 2000; Sluchanko & Gusev, 2010; Watanabe & Osada, 2016). This latter function is particularly relevant in light of the findings obtained in this study. As mentioned previously, a significant proportion of proteins exhibiting a higher level of t‐Cys, which are linked to aging and capable of interacting with 14‐3‐3 proteins as demonstrated by our in silico experiments, were observed to be involved in the organization of the cytoskeleton.

Previous reports suggested that t‐Cys can modify the structure and function of affected proteins, but the specific mechanism remains unclear (Garrido Ruiz et al., 2022). Our study provides the first evidence that t‐Cys mimics p‐Ser in terms of altering protein structure. This p‐Ser‐like modification mediated by t‐Cys may have highly similar implications in the regulation of protein function, though further exploration is required. Abnormal accumulation of proteins with altered levels of p‐Ser is known to be a core mechanism in several human pathologies, including Alzheimer's disease (AD), cancer, and other neurodegenerative conditions (Iqbal et al., 2013; Roesch et al., 2005; Yatsunami et al., 1993). Similarly, it has been clinically established that p‐Ser sites affecting structural proteins circulating in biological fluids, such as the microtubule protein Tau, serve as viable biological markers for diagnosing AD (Ashton et al., 2021; Ossenkoppele et al., 2022). Therefore, the potential capacity of t‐Cys sites as biological markers in aging‐associated diseases also warrants further investigation and holds promise for the upcoming clinical application of the findings reported.

### LIMITATIONS OF THE STUDY

We observed that the levels of p‐Ser residues identified in this study, when considering only highly confidently identified p‐Ser residues, were generally low. This is because the identification of p‐Ser residues typically involves phosphoproteome enrichment, which was not performed in this study due to its focus on the analysis of t‐Cys. Consequently, direct relative comparisons between the levels of these two post‐translational modifications could be partially compromised based on the generated data. Similarly, although we identified relevant associations in this study between specific enzymes, t‐Cys, and p‐Ser residues in the analyzed proteomes, further research is necessary to elucidate the biological significance of these findings. Finally, the findings encountered in this study, including the effect of biological sex on the t‐Cys proteome profiles in aging, require of further validation in larger cohort studies.

## AUTHOR CONTRIBUTIONS

S.K.S., A.S. and X.G‐P. contributed to the conceptualization, review and editing, funding acquisition, and supervision. J.A.S.M., M.F.‐R., G.X., M.M., S.C.N., R.I., and M.K. contributed to the experimental methodology. J.A.S.M. contributed to the manuscript draft. All authors have read and approved the submitted version.

## CONFLICT OF INTEREST STATEMENT

The authors declare that they have no competing interests with regard of the data and conclusions reported in this work. The interpretations provided are based on the obtained scientific data and are that of the authors and not necessarily that of the public bodies that funded the study.

## Supporting information


Data S1.

Data S2.

Data S3.

Data S4.

Figure S1.
Figure S2.
Figure S3.
Figure S4.
Figure S5.
Table S1.
Table S2.
Table S3.
Table S4.
Table S5.
Table S6.
Table S7.
Table S8.
Table S9.
Table S10.
Table S11.
Table S12.
Table S13.
Table S14.
Table S15.

## Data Availability

All proteomics data generated in this study have been made publicly available in ProteomeXchange through the specialized repository PRIDE with the identifier PXD037908. The scripts created for the bioinformatic analyses performed have also been made publicly available through Github: https://github.com/JoseASanchezMilan/tCys_project.git. The rest of the data generated have been included in supplementary files and remain at full disposition upon reasonable request.
