# Supplementary material for: Trioxidized cysteine in the aging proteome mimics the structural dynamics and interactome of phosphorylated serine
Source: Aging Cell. 2023 Dec 18;23(3):e14062. doi: 10.1111/acel.14062 (PMC10928580; doi:10.1111/acel.14062)
Supplement: Supplementary file 5 — Figure S1. Figure S2. Figure S3. Figure S4. Figure S5. Table S1. Table S2. Table S3. Table S4. Table S5. Table S6. Table S7. Table S8. Table S9. Table S10. Table S11. Table S12. Table S13. Table S14. Table S15. [file ACEL-23-e14062-s002.docx]

**Supplementary Materials**

**Trioxidized cysteine in aging mimic the dynamics and molecular interactome of protein phosphorylated serine**

Jose Antonio Sánchez Milán ^1, 2^, María Fernández-Rhodes^1, 2, ‡^, Xue Guo ^3, ‡^, María Mulet ^1, 2, ‡^, SoFong Cam Ngan ^4^, Ranjith Iyappan ^4^, Maryam Katoueezadeh ^4^, Siu Kwan Sze ^4, †,*^, Aida Serra ^1,†,*^ and Xavier Gallart-Palau ^2, 5, †,*^

^1^ Department of Basic Medical Sciences, University of Lleida (UdL) – Biomedical Research Institute of Lleida (IRB Lleida) - +Pec Proteomics Research Group (+PPRG) - Neuroscience Area, 25198 Lleida, Spain

^2^ Biomedical Research Institute of Lleida (IRBLLEIDA) - +Pec Proteomics Research Group (+PPRG) - Neuroscience Area – University Hospital Arnau de Vilanova (HUAV), 25198 Lleida, Spain

^3^ Institute of Molecular and Cell Biology (IMCB), 138673 Singapore.

^4^Department of Health Sciences, Faculty of Applied Health Sciences, Brock University, St. Catharines, Ontario L2S 3A1, Canada

^5^Department of Psychology, University of Lleida (UdL), 25198 Lleida, Spain

‡ María Fernández-Rhodes, Xue Guo and María Mulet contributed equally.

^†^Siu Kwan Sze, Aida Serra and Xavier Gallart-Palau are joint senior authors.

**Summary Table:**

| **Content** | **Page** |
| --- | --- |
| **Supplementary Table S1:** List of packages used for bioinformatic analysis. | S-3 |
| **Supplementary Table S2:** Descriptive statistics of the t-Cys stoichiometry calculated in the whole proteome. | S-4 |
| **Supplementary Table S3:** Modulation of t-Cys residues according to the variables sex and age. | S-5 |
| **Supplementary Table S4:** Proteins with higher levels of t-Cys in skin of aged mice. | S-6 |
| **Supplementary Table S5:** Proteins with higher levels of t-Cys in lungs of aged mice. | S-7 |
| **Supplementary Table S6:** Descriptive statistics of the t-Cys stoichiometry calculated for the proteins with significantly higher levels of t-Cys in old mice compared to young mice. | S-8 |
| **Supplementary Table S7:** Proteins with higher levels of p-Ser in skin of aged mice. | S-9 |
| **Supplementary Table S8:** Descriptive statistics of the p-Ser stoichiometry calculated in the whole proteome. | S-12 |
| **Supplementary Table S9:** Descriptive statistics of the p-Ser stoichiometry calculated for the proteins with significantly higher levels of p-Ser in old mice compared to young mice. | S-13 |
| **Supplementary Table S10:** Structural comparison of t-Cys and p-Ser. | S-14 |
| **Supplementary Table S11:** Predicted kinases that could interact with t-Cys and p-Ser sites. | S-15 |
| **Supplementary Table S12:** Docking scores of predicted kinases and the t-Cys and p-Ser sites. | S-17 |
| **Supplementary Table S13:** Study of the competitive binding between t-Cys and p-Ser. | S-19 |
| **Supplementary Table S14:** Interacting sites of CHUK and 14-3-3σ protein. | S-20 |
| **Supplementary Table S15:** Potential function of the modified sites from the age-dependent modulated proteins. | S-21 |
| **Supplementary Figure S1:** Detail of the t-Cys and p-Ser sites in ACTN2 and the distance between both modified residues. | S-22 |
| **Supplementary Figure S2:** t-Cys/p-Ser similarity index (TPSi). | S-23 |
| **Supplementary Figure S3:** Docking simulation of the kinases with the t-Cys and p-Ser sites. | S-24 |
| **Supplementary Figure S4:** Docking simulation of the 14-3-3σ protein with the t-Cys and p-Ser sites. | S-25 |
| **Supplementary Figure S5:** Cellular viability of HEK293 and SHSY5Y cells under oxidative stress conditions. | S-26 |
| **Supplementary Dataset 1:** List of identified proteins with t-Cys in skin dataset. | Additional file |
| **Supplementary Dataset 2:** List of identified proteins with t-Cys in lung dataset. | Additional file |
| **Supplementary Dataset 3:** List of identified proteins with p-Ser in skin dataset. | Additional file |
| **Supplementary Dataset 4:** List of identified proteins. | Additional file |

**Supplementary Table S1:** List of packages used for bioinformatic analysis.

| **Package** | **Version** |
| --- | --- |
| dplyr | 1.1.2 |
| biomaRt | 2.54.1 |
| edgeR | 3.40.2 |
| clusterProfiler | 4.6.2 |
| org.Mm.eg.db | 3.17.0 |
| tidyverse | 2.0.0 |
| corrplot | 0.92 |

**Supplementary Table S2:** Descriptive statistics of the t-Cys stoichiometry calculated at the proteome-wide level, in young and old animals. Young group includes 1-month and 6-months old animals while old group includes 18-months and 24-months old animals. The stoichiometry was calculated based on the spectral count of the peptides containing t-Cys compared to the spectral count of the unmodified counterparts.

|  | **Total** | | |  | **Young** | | |  | **Old** | | |
| --- | --- | --- | --- | --- | --- | --- | --- | --- | --- | --- | --- |
|  | **Non modified Cys Spectral Count** | **t-Cys Spectral Count** | **%**  **t-Cys** | | **Non modified Cys Spectral Count** | **t-Cys Spectral Count** | **%**  **t-Cys** | | **Non modified Cys Spectral Count** | **t-Cys Spectral Count** | **%**  **t-Cys** |
| **Min** | 0 | 5 | 0.42 | | 0 | 0 | 0 | | 0 | 0 | 0 |
| **1st Qu.** | 0 | 11 | 28.49 | | 1.75 | 0 | 0 | | 0.75 | 9 | 30.33 |
| **Median** | 17 | 27 | 67.77 | | 11 | 7.50 | 22.35 | | 13.50 | 24 | 67.71 |
| **Mean** | 84.80 | 47.68 | 63.32 | | 46.48 | 18.91 | 39.79 | | 54.66 | 36.92 | 60.33 |
| **3rd Qu.** | 60.25 | 52.50 | 100 | | 35.75 | 24.25 | 81.77 | | 39.25 | 45.25 | 97.32 |
| **Max.** | 3376 | 722 | 100 | | 1120 | 279 | 100 | | 2256 | 443 | 100 |

**Supplementary table S3:** Analysis of the effect of sex and age in t-Cys. The mean ± standard deviation of spectral count and number of t-Cys residues grouped by sex and age are displayed. Young group includes 1-month and 6-months old animals while old group includes 18-months and 24-months old animals. Significance was assessed by two-way ANOVA of the spectral counts and number of t-Cys, with a minimum significance level *p* < 0.05. Same letter in superscript indicates significant differences between conditions.

|  | **Female** | |  | **Male** | |  |
| --- | --- | --- | --- | --- | --- | --- |
|  | **Young** | **Old** | | **Young** | **Old** | **p-value** |
| **Spectral Count** | 286 ± 33.64^a^ | 1826.33 ± 185.08^b^ | | 620.67 ± 169.74^a^ | 1000.33 ± 21.55^b^ | 0,2624 |
| **Number of t-Cys** | 25.67 ± 0.57^a,b^ | 72.33 ± 1.53^a,c^ | | 49.33 ± 5.77^b^ | 74.67± 3.21^c^ | 0,0101 |

**Supplementary Table S4:** Trioxidized age-dependent modulated proteins identified in skin. The log2 of the fold change (log_2_FC), Fisher-test (F) and P-value from the differential expression analysis are displayed. Proteins are classified by its function according to UniprotDB and PantherDB. All the contrasts were performed comparing to the youngest group of 1 month and only significant differences towards this group have been included. Significance was assessed by differential expression analysis of the protein spectral counts, with a minimum significance level p< 0.05.

| **GS** | **Protein Description** | log_2_FC | | | **F** | **P-Value** |
| --- | --- | --- | --- | --- | --- | --- |
|  |  | **6 Months** | **18 Months** | **24 Months** |  |  |
| *Structural Organization* | | | | | | |
| Myh1 | Myosin-1 | -0.2647113 | 9.7989055 | 7.8569692 | 199.582639 | 0.0000000 |
| Krt17 | Keratin, type I cytoskeletal 17 | -0.2647113 | 0.7294112 | 4.8506238 | 24.154296 | 0.0000220 |
| Actn2 | Alpha-actinin-2 | 1.1131790 | 4.2338290 | 4.7791461 | 21.022402 | 0.0000444 |
| Sptbn1 | Spectrin beta chain, non-erythrocytic 1 | -0.2647113 | 1.1309889 | 4.0760328 | 9.918519 | 0.0014158 |
| Actbl2 | Beta-actin-like protein 2 | 0.9390956 | 1.7193700 | 3.9849545 | 7.162787 | 0.0051193 |
| Actn3 | Alpha-actinin-3 | -0.8579131 | 3.5398925 | 3.4631835 | 8.819787 | 0.0022894 |
| Krt5 | Keratin, type II cytoskeletal 5 | 0.7624518 | 0.8404180 | 0.7519246 | 3.490231 | 0.0498316 |
| Mylpf | Myosin regulatory light chain 2, skeletal muscle isoform | -4.8081958 | 0.6774770 | 0.5708066 | 66.810390 | 0.0000001 |
| *Cell Regulation / Epigenetics* | | | | | | |
| Gatd3a | Glutamine amidotransferase-like class 1 domain-containing protein 3A, mitochondrial | -0.2647113 | 0.1483065 | 1.5342480 | 4.418647 | 0.0258222 |
| Fhl1 | Four and a half LIM domains protein 1 | -0.2647113 | 2.6309094 | 1.5262275 | 9.355629 | 0.0018025 |
| *Cell Signaling / Kinases* | | | | | | |
| Ckm | Creatine kinase M-type | -0.2647113 | 2.0979559 | 4.4310745 | 23.481737 | 0.0000254 |
| Pgk1 | Phosphoglycerate kinase 1 | 2.1028788 | 2.9759214 | 3.2013222 | 7.492838 | 0.0043236 |
| *Metabolism* | | | | | | |
| Plin1 | Perilipin-1 | 1.1852859 | 2.3316189 | 4.6107579 | 28.709103 | 0.0000090 |
| Mdh2 | Malate dehydrogenase, mitochondrial | 1.0141239 | 3.5610598 | 4.0312233 | 13.727506 | 0.0003416 |
| Slc25a4 | ADP/ATP translocase 1 | 3.4992394 | 4.0055528 | 4.0108841 | 14.111279 | 0.0003010 |
| Aldoa | Fructose-bisphosphate aldolase A | -0.2647113 | 2.8647533 | 2.6072925 | 25.788442 | 0.0000157 |
| Acly | ATP-citrate synthase | -0.2647113 | 2.8997203 | 1.4641251 | 10.906025 | 0.0009474 |
| Aldh6a1 | Methylmalonate-semialdehyde dehydrogenase [acylating], mitochondrial | -0.2647113 | 4.0992735 | 0.2931855 | 27.739526 | 0.0000108 |
| *Immune Response* | | | | | | |
| Anxa1 | Annexin A1 | -0.2647113 | 3.3799501 | 3.4957813 | 58.074658 | 0.0000002 |
| *Cell Communication / Transport* | | | | | | |
| Vat1 | Synaptic vesicle membrane protein VAT-1 homolog | 0.7133761 | 2.3327439 | 3.8455140 | 8.338688 | 0.0028612 |

**Supplementary Table S5**: Trioxidized age-dependent modulated proteins identified in lung. The log2 of the fold change (log_2_FC), Fisher-test (F) and P-value from the differential expression analysis are displayed. Proteins are classified by its function according to UniprotDB and PantherDB. Significance was assessed by differential expression analysis of the protein spectral counts, with a minimum significance level *p* < 0.05.

| **GS** | **Protein Description** | **Old vs Young**  **log_2_FC** | **F** | **P-Value** |
| --- | --- | --- | --- | --- |
| *Structural Organization* | | | | |
| Myl7 | Myosin regulatory light chain 2,, atrial isoform | 2.9965354 | 5.311459 | 0.0197462 |
| Myh11 | Myosin-11 | 2.6945411 | 4.705654 | 0.0279897 |
| Myh10 | Myosin-10 | 2.5328797 | 4.967028 | 0.0240255 |
| Actg2 | Actin,, gamma-enteric smooth muscle | 2.4137188 | 5.166084 | 0.0214358 |
| Plec | Plectin | 2.2679190 | 5.434181 | 0.0184381 |
| Flna | Filamin-A | 2.2433314 | 5.484958 | 0.0179261 |
| Sptb | Spectrin beta chain,, erythrocytic | 1.9052636 | 6.273853 | 0.0117413 |
| Vcl | Vinculin | 1.8859225 | 5.910768 | 0.0142193 |
| Actn2 | Alpha-actinin-2 | 1.8185326 | 6.494501 | 0.0104785 |
| Tubb2b | Tubulin beta-2B chain | 1.6207752 | 6.563449 | 0.0101164 |
| *Metabolism* | | | | |
| Aldh1a7 | Aldehyde dehydrogenase,, cytosolic 1 | 1.6685679 | 6.866793 | 0.0086847 |
| Acat1 | Acetyl-CoA acetyltransferase,, mitochondrial | 1.5266238 | 6.772532 | 0.0091031 |
| Abca8a | ATP-binding cassette sub-family A member 8-A | 0.5134716 | 9.873849 | 0.0022468 |
| *Cell Regulation* | | | | |
| H2afy | Core histone macro-H2A.1 | 1.8348951 | 6.457428 | 0.0106794 |
| Rap1a | Ras-related protein Rap-1A | 1.3309388 | 7.584322 | 0.0061329 |
| Smarca5 | SWI/SNF-related matrix-associated actin-dependent regulator of chromatin subfamily A member 5 | 1.2201411 | 7.667712 | 0.0058966 |
| Podxl | Podocalyxin | 0.5546307 | 3.805344 | 0.0487069 |
| Terf2 | Telomeric repeat-binding factor 2 | 0.5134716 | 9.412858 | 0.0027186 |
| *Immune Response* | | | | |
| Selenbp1 | Methanethiol oxidase | 1.6352473 | 6.395975 | 0.0110221 |
| Scgb1a1 | Uteroglobin | 1.0462405 | 8.093211 | 0.0048419 |
| Igha | Immunoglobulin heavy constant alpha (Fragment) | 0.7105617 | 8.902083 | 0.0033796 |
| *Cell Signaling* | | | | |
| Ptpn1 | Tyrosine-protein phosphatase non-receptor type 1 | 1.6089526 | 9.429809 | 0.0026994 |
| *Cell Communication* | | | | |
| Tf | Serotransferrin | 4.5137629 | 8.223580 | 0.0045634 |
| Vdac3 | Voltage-dependent anion-selective channel protein 3 | 1.4518671 | 7.055440 | 0.0079118 |

**Supplementary Table S6:** Descriptive statistics of the t-Cys stoichiometry calculated for the trioxidized age-dependent modulated proteins. The stoichiometry was calculated based on the spectral count of the peptides containing t-Cys compared to the spectral count of the unmodified counterparts. Young group includes 1-month and 6-months old animals while old group includes 18-months and 24-months old animals. Table shows the minimum (Min), 1st quartile (1^st^ Q), median, mean, 3rd quartile (3^rd^ Q) and maximum value (Max).

|  | **Total^a^** | | |  | **Young^b^** | | |  | **Old^c^** | | |
| --- | --- | --- | --- | --- | --- | --- | --- | --- | --- | --- | --- |
|  | **Non modified Cys (Spectral Count)** | **t-Cys (Spectral Count)** | **%**  **t-Cys** | | **Non modified Cys (Spectral Count)** | **t-Cys (Spectral Count)** | **%**  **t-Cys** | | **Non modified Cys (Spectral Count)** | **t-Cys (Spectral Count)** | **%**  **t-Cys** |
| **Min** | 0 | 6 | 0.42 | | 0 | 0 | 0 | | 0 | 6 | 0.67 |
| **1^st^ Q** | 5.50 | 22.5 | 31.30 | | 3 | 0 | 0 | | 4 | 22.50 | 38.57 |
| **Median** | 21 | 39.5 | 66.61 | | 12 | 0 | 0 | | 14 | 34.50 | 73.54 |
| **Mean** | 162.24 | 57 | 60.91 | | 72.43 | 9.56 | 26.44 | | 103.98 | 49.30 | 64.74 |
| **3^rd^ Q** | 64.75 | 78 | 89.38 | | 35 | 10 | 62.5 | | 49.75 | 75.75 | 92.66 |
| **Max** | 3376 | 268 | 100 | | 1120 | 108 | 100 | | 2256 | 160 | 100 |

**Supplementary Table S7**: p-Ser age-dependent modulated proteins identified in skin. The log2 of the fold change (log_2_FC), Fisher-test (F) and P-value from the differential expression analysis are displayed. All the contrast were performed comparting with the youngest group of 1 moth. Significance was assessed by differential expression analysis of the protein spectral counts, with a minimum significance level p< 0.05.

| **GS** | **Protein Description** | log_2_FC | | | **F** | **P-Value** |
| --- | --- | --- | --- | --- | --- | --- |
|  |  | **6 Months** | **18 Months** | **24 Months** |  |  |
| Dnaja2 | DnaJ homolog subfamily A member 2 | -0.7305783 | 3.2031214 | 1.9079944 | 13.541101 | 0.0007604 |
| Myh8 | Myosin-8 | 1.2147855 | 2.0724463 | 1.7324732 | 20.393313 | 0.0001434 |
| Myh4 | Myosin-4 | 0.7389038 | 2.1259345 | 1.5753954 | 21.208207 | 0.0001215 |
| Myh1 | Myosin-1 | 0.7389038 | 2.4072484 | 1.5753954 | 32.400155 | 0.0000191 |
| Plec | Plectin | 2.2868023 | 0.4658930 | 1.3535555 | 6.928641 | 0.0084511 |
| Myh7 | Myosin-7 | 1.0892200 | 2.0907480 | 1.2618197 | 11.405908 | 0.0014696 |
| Myh6 | Myosin-6 | 0.8293521 | 1.7636199 | 1.2618197 | 9.943183 | 0.0024408 |
| Myh3 | Myosin-3 | 0.5803484 | 2.7039748 | 1.1167928 | 15.772739 | 0.0004145 |
| Krt6a | Keratin type II cytoskeletal 6A | 0.5815314 | 1.5225346 | 1.1006056 | 4.964732 | 0.0232511 |
| Gnb1 | Guanine nucleotide-binding protein G(I)/G(S)/G(T) subunit beta-1 | 2.3763512 | 2.0138450 | 1.1006056 | 20.857689 | 0.0001304 |
| Krt1 | Keratin type II cytoskeletal 1 | -1.0040677 | 0.1837737 | 0.9440361 | 9.795136 | 0.0025771 |
| Mre11a | Double-strand break repair protein MRE11 | 1.6394806 | 0.9942138 | 0.5799993 | 5.788118 | 0.0148399 |
| Lama2 | Laminin subunit alpha-2 | 1.6394806 | 0.4658930 | 0.5799993 | 6.445523 | 0.0106462 |
| Krt84 | Keratin type II cuticular Hb4 | 0.0547746 | 1.5225346 | 0.5799993 | 16.239859 | 0.0003683 |
| Krt8 | Keratin type II cytoskeletal 8 | 0.0547746 | 1.5225346 | 0.5799993 | 16.239859 | 0.0003683 |
| Krt79 | Keratin type II cytoskeletal 79 | 0.5815314 | 1.5225346 | 0.5799993 | 5.592426 | 0.0164532 |
| Krt77 | Keratin type II cytoskeletal 1b | 0.0547746 | 2.2595002 | 0.5799993 | 42.179857 | 0.0000058 |
| Krt76 | Keratin type II cytoskeletal 2 oral | 0.0547746 | 1.7681898 | 0.5799993 | 16.602086 | 0.0003367 |
| Krt75 | Keratin type II cytoskeletal 75 | 0.5815314 | 1.5225346 | 0.5799993 | 5.592426 | 0.0164532 |
| Krt73 | Keratin type II cytoskeletal 73 | 0.0547746 | 1.5225346 | 0.5799993 | 16.239859 | 0.0003683 |
| Krt72 | Keratin type II cytoskeletal 72 | 0.5869130 | 1.5225346 | 0.5799993 | 5.890725 | 0.0140698 |
| Krt7 | Keratin type II cytoskeletal 7 | 0.0547746 | 1.5225346 | 0.5799993 | 16.239859 | 0.0003683 |
| Krt5 | Keratin type II cytoskeletal 5 | 0.0547746 | 1.5225346 | 0.5799993 | 16.239859 | 0.0003683 |
| Krt4 | Keratin type II cytoskeletal 4 | 0.0547746 | 1.5225346 | 0.5799993 | 16.239859 | 0.0003683 |
| Krt2 | Keratin type II cytoskeletal 2 epidermal | 0.0547746 | 1.5225346 | 0.5799993 | 16.239859 | 0.0003683 |
| Krt14 | Keratin type I cytoskeletal 14 | 1.1149339 | 1.5225346 | 0.5642338 | 5.121921 | 0.0212750 |
| Matr3 | Matrin-3 | 0.8707276 | 0.4329388 | 0.5336615 | 5.287300 | 0.0194086 |
| Kmt2d | Histone-lysine N-methyltransferase 2D | 0.0551744 | 0.1001324 | 0.5243766 | 6.251312 | 0.0117174 |
| Vim | Vimentin | 0.0547746 | 0.9942138 | 0.0405277 | 4.406716 | 0.0322867 |
| Tpm1 | Tropomyosin alpha-1 chain | 0.0547746 | 1.5225346 | 0.0405277 | 110.383017 | 0.0000001 |
| Tpi1 | Triosephosphate isomerase | 0.0547746 | 0.9942138 | 0.0405277 | 4.406716 | 0.0322867 |
| Tmpo | Lamina-associated polypeptide 2 isoforms beta/delta/epsilon/gamma | 0.0547746 | 0.9942138 | 0.0405277 | 4.406716 | 0.0322867 |
| Steap4 | Metalloreductase STEAP4 | 1.6394806 | 0.4658930 | 0.0405277 | 14.931511 | 0.0005166 |
| Serpina3b | Serine protease inhibitor A3B | 0.0547746 | 0.9942138 | 0.0405277 | 4.406716 | 0.0322867 |
| Selenoo | Protein adenylyltransferase SelO mitochondrial | 0.0547746 | 0.9942138 | 0.0405277 | 4.406716 | 0.0322867 |
| Scrib | Protein scribble homolog | 0.0547746 | 0.9942138 | 0.0405277 | 4.406716 | 0.0322867 |
| Pygm | Glycogen phosphorylase muscle form | 0.0547746 | 0.9942138 | 0.0405277 | 4.406716 | 0.0322867 |
| Pvalb | Parvalbumin alpha | 0.0547746 | 1.5225346 | 0.0405277 | 110.383017 | 0.0000001 |
| Prpsap2 | Phosphoribosyl pyrophosphate synthase-associated protein 2 | 0.0547746 | 0.9942138 | 0.0405277 | 4.406716 | 0.0322867 |
| Ppia | Peptidyl-prolyl cis-trans isomerase A | 0.0547746 | 1.7681898 | 0.0405277 | 46.554863 | 0.0000037 |
| Polr2a | DNA-directed RNA polymerase II subunit RPB1 | 0.0547746 | 0.9942138 | 0.0405277 | 4.406716 | 0.0322867 |
| Plrg1 | Pleiotropic regulator 1 | 0.0547746 | 0.9942138 | 0.0405277 | 4.406716 | 0.0322867 |
| Plcd1 | 1-phosphatidylinositol 4 5-bisphosphate phosphodiesterase delta-1 | 0.5869130 | 1.5225346 | 0.0405277 | 12.781220 | 0.0009519 |
| Npm1 | Nucleophosmin | 0.0547746 | 0.9942138 | 0.0405277 | 4.406716 | 0.0322867 |
| Myl1 | Myosin light chain 1/3 skeletal muscle isoform | 0.0547746 | 1.7681898 | 0.0405277 | 46.554863 | 0.0000037 |
| Lum | Lumican | 0.0547746 | 0.9942138 | 0.0405277 | 4.406716 | 0.0322867 |
| Krt42 | Keratin type I cytoskeletal 42 | 1.1149339 | 1.5225346 | 0.0405277 | 12.060674 | 0.0011895 |
| Krt17 | Keratin type I cytoskeletal 17 | 1.1149339 | 1.5225346 | 0.0405277 | 12.060674 | 0.0011895 |
| Itih1 | Inter-alpha-trypsin inhibitor heavy chain H1 | 0.0547746 | 0.9942138 | 0.0405277 | 4.406716 | 0.0322867 |
| Idh3a | Isocitrate dehydrogenase [NAD] subunit alpha mitochondrial | 0.0547746 | 0.9942138 | 0.0405277 | 4.406716 | 0.0322867 |
| Hnrnpab | Heterogeneous nuclear ribonucleoprotein A/B | 0.0547746 | 0.9942138 | 0.0405277 | 4.406716 | 0.0322867 |
| Gc | Vitamin D-binding protein | 1.1189267 | 0.9942138 | 0.0405277 | 3.731670 | 0.0494892 |
| Fn1 | Fibronectin | 0.0547746 | 0.9942138 | 0.0405277 | 4.406716 | 0.0322867 |
| Fhod1 | FH1/FH2 domain-containing protein 1 | 0.0547746 | 1.5225346 | 0.0405277 | 110.383017 | 0.0000001 |
| Fam120a | Constitutive coactivator of PPAR-gamma-like protein 1 | 1.8837987 | 0.4658930 | 0.0405277 | 15.350249 | 0.0004624 |
| Eno1b | Alpha-enolase | 0.0547746 | 1.5225346 | 0.0405277 | 110.383017 | 0.0000001 |
| Eno1 | Alpha-enolase | 0.0547746 | 1.5225346 | 0.0405277 | 110.383017 | 0.0000001 |
| Dkc1 | H/ACA ribonucleoprotein complex subunit DKC1 | 0.0547746 | 1.2398690 | 0.0405277 | 4.146661 | 0.0379048 |
| Dcn | Decorin | 0.5803484 | 2.9866404 | 0.0405277 | 50.011881 | 0.0000026 |
| Cox4i1 | Cytochrome c oxidase subunit 4 isoform 1 mitochondrial | 0.0547746 | 0.9942138 | 0.0405277 | 4.406716 | 0.0322867 |
| Chdh | Choline dehydrogenase mitochondrial | 0.0547746 | 0.9942138 | 0.0405277 | 4.406716 | 0.0322867 |
| Ccbe1 | Collagen and calcium-binding EGF domain-containing protein 1 | 0.0547746 | 0.9942138 | 0.0405277 | 4.406716 | 0.0322867 |
| Atp5a1 | ATP synthase subunit alpha mitochondrial | 0.0547746 | 2.7449270 | 0.0405277 | 456.071812 | 0.0000000 |
| Atl3 | Atlastin-3 | 0.0547746 | 0.9942138 | 0.0405277 | 4.406716 | 0.0322867 |
| Actn2 | Alpha-actinin-2 | 0.0547746 | 1.5225346 | 0.0405277 | 110.383017 | 0.0000001 |
| Actn1 | Alpha-actinin-1 | 1.0988921 | 1.5225346 | 0.0405277 | 10.748786 | 0.0018341 |

**Supplementary Table S8:** Descriptive statistics of the p-Ser stoichiometry calculated at proteome-wide level, in the young group and the old groups. Young group includes 1-month and 6-months old animals while old group includes 18-months and 24-months old animals. The stoichiometry was calculated based on the spectral count of the peptides containing p-Ser compared to the spectral count of the unmodified counterparts.

|  | **Total** | | |  | **Young** | | |  | **Old** | | |
| --- | --- | --- | --- | --- | --- | --- | --- | --- | --- | --- | --- |
|  | **Non modified Ser (Spectral Count)** | **p-Ser (Spectral Count)** | **% p-Ser** | | **Non modified Ser (Spectral Count)** | **p-Ser (Spectral Count)** | **% p-Ser** | | **Non modified Ser (Spectral Count)** | **p-Ser (Spectral Count)** | **% p-Ser** |
| **Min** | 0 | 0 | 0 | | 0 | 0 | 0 | | 0 | 0 | 0 |
| **1st Qu.** | 0 | 1 | 100 | | 0 | 1 | 100 | | 0 | 1 | 66.67 |
| **Median** | 0 | 1 | 100 | | 0 | 1 | 100 | | 0 | 1 | 100 |
| **Mean** | 30.09 | 3.78 | **85.46** | | 21.68 | 3.01 | **79.05** | | 27.43 | 3.17 | **77.19** |
| **3rd Qu.** | 0 | 2 | 100 | | 0 | 2 | 100 | | 1 | 2 | 100 |
| **Max.** | 5495 | 422 | 100 | | 2574 | 422 | 100 | | 3120 | 204 | 100 |

**Supplementary Table S9:** Descriptive statistics of the p-Ser stoichiometry calculated for the p-Ser age-dependent modulated proteins. The stoichiometry was calculated based on the spectral count of the peptides containing p-Ser compared to the spectral count of the unmodified counterparts. Young group includes 1-month and 6-months old animals while old group includes 18-months and 24-months old animals. Table shows the minimum (Min), 1st quartile (1^st^ Q), median, mean, 3rd quartile (3^rd^ Q) and maximum value (Max).

|  | **Total** | | | |  | **Young** | | |  | **Old** | | |
| --- | --- | --- | --- | --- | --- | --- | --- | --- | --- | --- | --- | --- |
|  | **Non modified Ser Spectral Count** | | **p-Ser Spectral Count** | **%**  **p-Ser** | | **Non modified Ser Spectral Count** | **p-Ser Spectral Count** | **%**  **p-Ser** | | **Non modified Ser Spectral Count** | **p-Ser Spectral Count** | **%**  **p-Ser** |
| **Min** | | 0 | 0 | 0 | | 0 | 0 | 0 | | 0 | 0 | 0 |
| **1st Q** | | 0 | 2 | 1.43 | | 0 | 0 | 0 | | 0 | 1 | 1.92 |
| **Median** | | 2 | 3 | 66.66 | | 17.5 | 0 | 0 | | 2 | 2 | 32.43 |
| **Mean** | | 290.6 | 8.804 | 53.21 | | 175.4 | 3.01 | 34.03 | | 181.8 | 7.29 | 49.56 |
| **3rd Q** | | 156 | 7 | 100 | | 146.5 | 2.25 | 100 | | 99 | 6 | 100 |
| **Max** | | 5495 | 132 | 100 | | 2315 | 54 | 100 | | 3120 | 80 | 100 |

**Supplementary Table S10:** Structural comparison of t-Cys and p-Ser affected proteins. For the structural similarity of t-Cys and p-Ser the RSMD values of fragments with cysteic acid modification for t-Cys and phosphoserine substitution for p-Ser were obtained by using the sequence fragments with no modification as reference in VMD software. After performing MD simulation, the Root Mean Square Deviation values (RSMD) were obtained by GROMACS software and VMD trajectory tool, adjusting the calculation of the RMSD values by Backbone. Mean RSMD and the SD are expressed in Armstrong (Å). t-Cys vs. p-Ser similarity index (TPSi) reflect the similarities of the structures and were obtained by applying the TPSi function. Significance was assessed by performing one way ANOVA and Tukey’s test, with a minimum significance level p< 0.05.

| **GS** | **Protein Description** | **PTM site** | **RSMD t-Cys** | **RSMD**  **p-Ser** | **Software** | **RSMD traject t-Cys** | **RSMD traject p-Ser** | **TPSi** | **P-value** |
| --- | --- | --- | --- | --- | --- | --- | --- | --- | --- |
| Actn2 | Alpha-actinin-2 | 339 | 3,110 | 3,762 | VMD | 10,5 ± 0,651 | 10,31 ± 0.598 | 0,944 | 0,2084 |
| Krt5 | Keratin, type II cytoskeletal 5 | 52 | 2,952 | 3,654 | GROMACS | 6,675 ± 1,723 | 6,198 ± 2,896 | 0,811 | 0,2198 |
| Krt5 | Keratin, type II cytoskeletal 5 | 401 | 3,064 | 3,652 | VMD | 9,823 ± 0,522 | 9,91 ± 0,547 | 0,998 | 0,7616 |
| Krt17 | Keratin, type I cytoskeletal 17 | 29 | 2,926 | 3,614 | GROMACS | 6,592 ± 2,002 | 6,621 ± 2,003 | 0,922 | 0,9931 |
| Myh1 | Myosin-1 | 679 | 3,057 | 3,601 | GROMACS | 3,580 ± 0,753 | 3,877 ± 0,899 | 0,885 | 0,1022 |
| Myh1 | Myosin-1 | 1347 | 3,707 | 4,523 | VMD | 6,701 ± 0,647 | 6,452 ± 0,654 | 0,891 | 0,0823 |
| Myh1 | Myosin-1 | 1418 | 3,447 | 4,161 | VMD | 1,274 ± 0,337 | 1,233 ± 0,415 | 0,922 | 0,7751 |

**Supplementary Table S11.** Predicted kinases that could interact with t-Cys and p-Ser sites. For every protein the Gene Symbol (GS), Protein description, PTM site and modification and the Predicted kinase score are displayed. Scores were obtained using GPS 5.0 software for the fragments with the PTM site ±10 amino acids. The kinases with the highest scores for every PTM site are included.

| **Protein GS** | **Protein Description** | **Modified Site** | **PTM** | **Predicted Kinase GS** | **Kinase Description** | **Predicted Kinase Score** |
| --- | --- | --- | --- | --- | --- | --- |
| Actn2 | Alpha-actinin-2 | 339 | t-Cys | CAMK2D | Calcium/calmodulin-dependent protein kinase II, delta | 63.807 |
|  |  | 339 | t-Cys | CHUK | Inhibitor of nuclear factor kappa-B kinase | 224.057 |
|  |  | 624 | p-Ser | CHUK | Inhibitor of nuclear factor kappa-B kinase | 253.238 |
|  |  | 760 | p-Ser | CHUK | Inhibitor of nuclear factor kappa-B kinase | 262.32 |
|  |  | 624 | p-Ser | GRK2 | G protein-coupled receptor kinase 2 | 148.125 |
|  |  | 624 | p-Ser | PRKCG | Protein kinase C, gamma | 121.733 |
|  |  | 187 | t-Cys | PRKCG | Protein kinase C, gamma | 120.488 |
|  |  | 760 | p-Ser | PRKCG | Protein kinase C, gamma | 147.36 |
|  |  | 50 | p-Ser | PRKCG | Protein kinase C, gamma | 117.112 |
|  |  | 339 | t-Cys | PRKCH | Protein kinase C, eta | 40.45 |
|  |  | 187 | t-Cys | PRKCZ | Protein kinase C, zeta | 42.315 |
|  |  | 50 | p-Ser | SRPK | Serine-arginine protein kinase | 362.342 |
|  |  | 760 | p-Ser | STK3 | Serine/threonine kinase 3 | 90.828 |
|  |  | 187 | t-Cys | TK | Tyrosine kinase | 49.893 |
|  |  | 50 | p-Ser | VRK2 | Vaccinia related kinase 2 | 63.592 |
| Krt17 | Keratin, type I cytoskeletal 17 | 182 | p-Ser | CAMK2D | Calcium/calmodulin-dependent protein kinase II, delta | 64.889 |
|  |  | 182 | p-Ser | CHUK | Inhibitor of nuclear factor kappa-B kinase | 231.807 |
|  |  | 29 | t-Cys | CHUK | Inhibitor of nuclear factor kappa-B kinase | 281.622 |
|  |  | 29 | t-Cys | CLK | CDC-like kinase | 86.766 |
|  |  | 29 | t-Cys | PRKCG | Protein kinase C, gamma | 121.17 |
|  |  | 182 | p-Ser | STK3 | Serine/threonine kinase 3 | 85.002 |
| Krt5 | Keratin, type II cytoskeletal 5 | 401 | t-Cys | CHUK | Inhibitor of nuclear factor kappa-B kinase | 225.039 |
|  |  | 175 | p-Ser | CHUK | Inhibitor of nuclear factor kappa-B kinase | 336.497 |
|  |  | 52 | t-Cys | CSNK1E | Casein kinase 1, epsilon | 45.049 |
|  |  | 401 | t-Cys | IRAK1 | Interleukin-1 receptor-associated kinase 1 | 104.16 |
|  |  | 401 | t-Cys | PRKCG | Protein kinase C, gamma | 122.722 |
|  |  | 175 | p-Ser | PRKCG | Protein kinase C, gamma | 124.943 |
|  |  | 52 | t-Cys | PRKCZ | Protein kinase C, zeta | 55.527 |
|  |  | 175 | p-Ser | PRKCZ | Protein kinase C, zeta | 59.685 |
|  |  | 52 | t-Cys | VRK2 | Vaccinia related kinase 2 | 54.733 |
| Myh1 | Myosin-1 | 1418 | t-Cys | AURKA | Aurora kinase A | 50.158 |
|  |  | 1783 | p-Ser | BARK | Beta adrenergic receptor kinase | 165.934 |
|  |  | 1577 | p-Ser | CAMK2D | Calcium/calmodulin-dependent Protein kinase II, delta | 57.597 |
|  |  | 820 | t-Cys | CAMK2D | Calcium/calmodulin-dependent Protein kinase II, delta | 63.732 |
|  |  | 712 | t-Cys | CAMK2D | Calcium/calmodulin-dependent Protein kinase II, delta | 54.148 |
|  |  | 702 | t-Cys | CHUK | Inhibitor of nuclear factor kappa-B kinase | 264.804 |
|  |  | 820 | t-Cys | CHUK | Inhibitor of nuclear factor kappa-B kinase | 242.079 |
|  |  | 1135 | p-Ser | CHUK | Inhibitor of nuclear factor kappa-B kinase | 226.169 |
|  |  | 1418 | t-Cys | CHUK | Inhibitor of nuclear factor kappa-B kinase | 236.98 |
|  |  | 679 | t-Cys | CHUK | Inhibitor of nuclear factor kappa-B kinase | 266.285 |
|  |  | 1373 | p-Ser | CHUK | Inhibitor of nuclear factor kappa-B kinase | 281.068 |
|  |  | 1923 | p-Ser | CHUK | Inhibitor of nuclear factor kappa-B kinase | 286.36 |
|  |  | 1206 | p-Ser | CHUK | Inhibitor of nuclear factor kappa-B kinase | 293.172 |
|  |  | 1577 | p-Ser | CHUK | Inhibitor of nuclear factor kappa-B kinase | 289.717 |
|  |  | 1639 | p-Ser | CHUK | Inhibitor of nuclear factor kappa-B kinase | 228.678 |
|  |  | 1347 | t-Cys | CHUK | Inhibitor of nuclear factor kappa-B kinase | 291.905 |
|  |  | 1880 | p-Ser | CHUK | Inhibitor of nuclear factor kappa-B kinase | 254.583 |
|  |  | 712 | t-Cys | ILK | Integrin linked kinase | 27.462 |
|  |  | 712 | t-Cys | ILK | Integrin linked kinase | 27.462 |
|  |  | 1373 | p-Ser | IRAK1 | Interleukin-1 receptor-associated kinase 1 | 104.48 |
|  |  | 1206 | p-Ser | IRAK1 | Interleukin-1 receptor-associated kinase 1 | 107.845 |
|  |  | 1135 | p-Ser | IRAK1 | Interleukin-1 receptor-associated kinase 1 | 89.167 |
|  |  | 1347 | t-Cys | IRAK1 | Interleukin-1 receptor-associated kinase 1 | 92.707 |
|  |  | 1923 | p-Ser | NEK | NIMA-related kinase | 120.444 |
|  |  | 679 | t-Cys | NEK | NIMA-related kinase | 123.756 |
|  |  | 1880 | p-Ser | PRKCG | Protein kinase C, gamma | 158.121 |
|  |  | 1577 | p-Ser | PRKCG | Protein kinase C, gamma | 144.508 |
|  |  | 1923 | p-Ser | PRKCG | Protein kinase C, gamma | 132.581 |
|  |  | 1373 | p-Ser | PRKCG | Protein kinase C, gamma | 117.925 |
|  |  | 1206 | p-Ser | PRKCG | Protein kinase C, gamma | 119.846 |
|  |  | 1135 | p-Ser | PRKCG | Protein kinase C, gamma | 132.03 |
|  |  | 1347 | t-Cys | PRKCG | Protein kinase C, gamma | 131.564 |
|  |  | 1418 | t-Cys | PRKCG | Protein kinase C, gamma | 119.492 |
|  |  | 679 | t-Cys | PRKCG | Protein kinase C, gamma | 124.99 |
|  |  | 702 | t-Cys | PRKCG | Protein kinase C, gamma | 130.797 |
|  |  | 1639 | p-Ser | PRKCG | Protein kinase C, gamma | 145.254 |
|  |  | 1783 | p-Ser | PRKCH | Protein kinase C, eta | 37.876 |
|  |  | 1783 | p-Ser | PRKCZ | Protein kinase C, zeta | 56.701 |
|  |  | 1639 | p-Ser | PRKCZ | Protein kinase C, zeta | 60.528 |
|  |  | 702 | t-Cys | PRKCZ | Protein kinase C, zeta | 57.523 |
|  |  | 1880 | p-Ser | STK3 | Serine/threonine kinase 3 | 82.197 |
|  |  | 820 | t-Cys | STK3 | Serine/threonine kinase 3 | 75.381 |

**Supplementary Table S12:** Docking scores of predicted kinases and the t-Cys and p-Ser sites. Docking template score is the docking score resulting from the simulation of the fragment with no PTM with the corresponding kinase and Docking PTM score is the score with the cisteic acid substitution in t-Cys or phosphoserine in p-Ser. Docking scores were obtained using the Hdock server uploading the PDB structures.

| **Protein GS** | **Protein Description** | **Modified Site** | **PTM** | **Predicted Kinase GS** | **Kinase Description** | **Docking Template Score** | **Docking PTM Score** |
| --- | --- | --- | --- | --- | --- | --- | --- |
| Krt5 | Keratin, type II cytoskeletal 5 | 52 | t-Cys | PRKCZ | Protein kinase C, zeta | - | 0.7522 |
| Myh1 | Myosin-1 | 702 | t-Cys | PRKCZ | Protein kinase C, zeta | - | 0.7368 |
| Krt5 | Keratin, type II cytoskeletal 5 | 175 | p-Ser | PRKCZ | Protein kinase C, zeta | 0.7236 | 0.7034 |
| Myh1 | Myosin-1 | 1783 | p-Ser | PRKCZ | Protein kinase C, zeta | 0.5693 | 0.5652 |
| Actn2 | Alpha-actinin-2 | 187 | t-Cys | PRKCG | Protein kinase C, gamma | - | 0.8392 |
| Actn2 | Alpha-actinin-2 | 50 | p-Ser | PRKCG | Protein kinase C, gamma | 0.8176 | 0.8178 |
| Actn2 | Alpha-actinin-2 | 761 | p-Ser | PRKCG | Protein kinase C, gamma | 0.8154 | 0.7925 |
| Actn2 | Alpha-actinin-2 | 624 | p-Ser | PRKCG | Protein kinase C, gamma | 0.7307 | 0.6589 |
| Myh1 | Myosin-1 | 1373 | p-Ser | CHUK | Inhibitor of nuclear factor kappa-B kinase | 0.9961 | 0.9943 |
| Myh1 | Myosin-1 | 1922 | p-Ser | CHUK | Inhibitor of nuclear factor kappa-B kinase | 0.9894 | 0.9854 |
| Actn2 | Alpha-actinin-2 | 761 | p-Ser | CHUK | Inhibitor of nuclear factor kappa-B kinase | 0.8830 | 0.8112 |
| Myh1 | Myosin-1 | 679 | t-Cys | CHUK | Inhibitor of nuclear factor kappa-B kinase | - | 0.8266 |
| Myh1 | Myosin-1 | 820 | t-Cys | CHUK | Inhibitor of nuclear factor kappa-B kinase | - | 0.8768 |
| Actn2 | Alpha-actinin-2 | 339 | t-Cys | CHUK | Inhibitor of nuclear factor kappa-B kinase | - | 0.8819 |
| Myh1 | Myosin-1 | 1639 | p-Ser | CHUK | Inhibitor of nuclear factor kappa-B kinase | 0.8190 | 0.8248 |
| Myh1 | Myosin-1 | 702 | t-Cys | CHUK | Inhibitor of nuclear factor kappa-B kinase | - | 0.7444 |
| Myh1 | Myosin-1Myosin-1 | 1347 | t-Cys | CHUK | Inhibitor of nuclear factor kappa-B kinase | - | 0.7292 |
| Krt5 | Krt5 Keratin, type II cytoskeletal 5 | 175 | p-Ser | CHUK | Inhibitor of nuclear factor kappa-B kinase | 0.7811 | 0.7386 |
| Actn2 | Alpha-actinin-2 | 624 | p-Ser | CHUK | Inhibitor of nuclear factor kappa-B kinase | 0.7773 | 0.7545 |
| Krt17 | Keratin, type I cytoskeletal 17 | 29 | t-Cys | CHUK | Inhibitor of nuclear factor kappa-B kinase | - | 0.7476 |
| Krt17 | Keratin, type I cytoskeletal 17 | 182 | p-Ser | CHUK | Inhibitor of nuclear factor kappa-B kinase | 0.7632 | 0.6687 |
| Myh1 | Myosin-1 | 1206 | p-Ser | CHUK | Inhibitor of nuclear factor kappa-B kinase | 0.7524 | 0.7474 |
| Myh1 | Myosin-1 | 1880 | p-Ser | CHUK | Inhibitor of nuclear factor kappa-B kinase | 0.7133 | 0.6682 |
| Krt5 | Krt5 Keratin, type II cytoskeletal 5 | 401 | t-Cys | CHUK | Inhibitor of nuclear factor kappa-B kinase | - | 0.6980 |
| Myh1 | Myosin-1 | 1418 | t-Cys | CHUK | Inhibitor of nuclear factor kappa-B kinase | - | 0.6734 |
| Myh1 | Myosin-1 | 1577 | p-Ser | CHUK | Inhibitor of nuclear factor kappa-B kinase | 0.6475 | 0.6791 |
| Myh1 | Myosin-1 | 1135 | p-Ser | CHUK | Inhibitor of nuclear factor kappa-B kinase | 0.5975 | 0.5324 |
| Myh1 | Myosin-1 | 820 | t-Cys | CAMK2D | Calcium/calmodulin-dependent protein kinase II, delta | - | 0.8456 |
| Myh1 | Myosin-1 | 712 | t-Cys | CAMK2D | Calcium/calmodulin-dependent protein kinase II, delta | - | 0.8227 |
| Myh1 | Myosin-1 | 1577 | p-Ser | CAMK2D | Calcium/calmodulin-dependent protein kinase II, delta | 0.7058 | 0.6626 |

**Supplementary Table S13:** Study of the competitive binding between CHUK kinase and p-Ser and t-Cys residues when they are closely located within the primary structure of the affected proteins. The probability to interact between CHUK kinase and Myh1 regions containing p-Ser, t-Cys or both modified residues (p-Ser/t-Cys) is expressed as the percentage of the top10 docking models with docking score higher than 0.7.

| **Protein** | **p-Ser** | **t-Cys** | **p-Ser/t-Cys** |
| --- | --- | --- | --- |
| Myh1  1347t-Cys-1373p-Ser | 40% | 70% | 20% |

**Supplementary table S14**: Interacting sites of CHUK and 14-3-3σ protein identified by docking simulations with the t-Cys and p-Ser residues contained in the age-dependent modulated proteins. The domain or function exerted by the interacting sites in the protein have been obtained from Uniprot and InterPro.

| **Gene Symbol** | **Interacting Site Position** | **Residue** | **Domain/Function** |
| --- | --- | --- | --- |
| CHUK | 460 | E | Leucine-zipper  Scaffold dimerization domain |
|  | 464 | L | Leucine-zipper  Scaffold dimerization domain |
|  | 482 | F | Scaffold dimerization domain |
|  | 497 | Q | Scaffold dimerization domain |
|  | 500 | Y | Scaffold dimerization domain |
|  | 654 | L | Scaffold dimerization domain |
|  | 657 | A | Scaffold dimerization domain |
|  | 740 | W | NEMO binding |
|  | 742 | W | NEMO binding |
| 14-3-3σ | 49 | K | Peptide binding site |
|  | 56 | R | Interaction with phosphoserine |
|  | 130 | Y | Peptide binding site |
|  | 178 | V | Peptide binding site |
|  | 182 | E | Peptide binding site |
|  | 226 | N | Peptide binding site |
|  | 230 | W | Peptide binding site |

**Supplementary table S15:** Potential function of the sites from the age-dependent modulated proteins affected by the post-translational modifications (PTM) t-Cys and p-Ser. The domain or function exerted by the sites that undergo t-Cys or p-Ser modifications is provided based on Uniprot and InterPro information.

| **Protein** | **Modified site position** | **PTM** | **Domain/Function** |
| --- | --- | --- | --- |
| Myh1 | 679 | t-Cys | Actin Binding  Myosin motor |
|  | 702 | t-Cys | Myosin motor |
|  | 1347 | t-Cys | Coiled coil |
|  | 1418 | t-Cys | Coiled coil |
|  | 1135 | p-Ser | Coiled coil |
|  | 1206 | p-Ser | Coiled coil |
|  | 1373 | p-Ser | Coiled coil |
|  | 1577 | p-Ser | Coiled coil |
|  | 1639 | p-Ser | Coiled coil |
|  | 1783 | p-Ser | Coiled coil |
|  | 1922 | p-Ser | Coiled coil |
| Krt5 | 52 | t-Cys | Head |
|  | 401 | t-Cys | Coil 2  IF rod Domain |
|  | 175 | p-Ser | Coil 1A  IF rod Domain |
| Krt17 | 29 | t-Cys | Head |
|  | 182 | p-Ser | Coil 1b  IF rod Domain |
| Actn2 | 187 | t-Cys | Actin-binding  Calponin-homology (CH) 2 Domain |
|  | 339 | t-Cys | Spectrin 1 |
|  | 50 | p-Ser | Actin-binding  Calponin-homology (CH) 1 Domain |
|  | 624 | p-Ser | Spectrin 3 |
|  | 769 | p-Ser | EF hand 1 Domain |

*
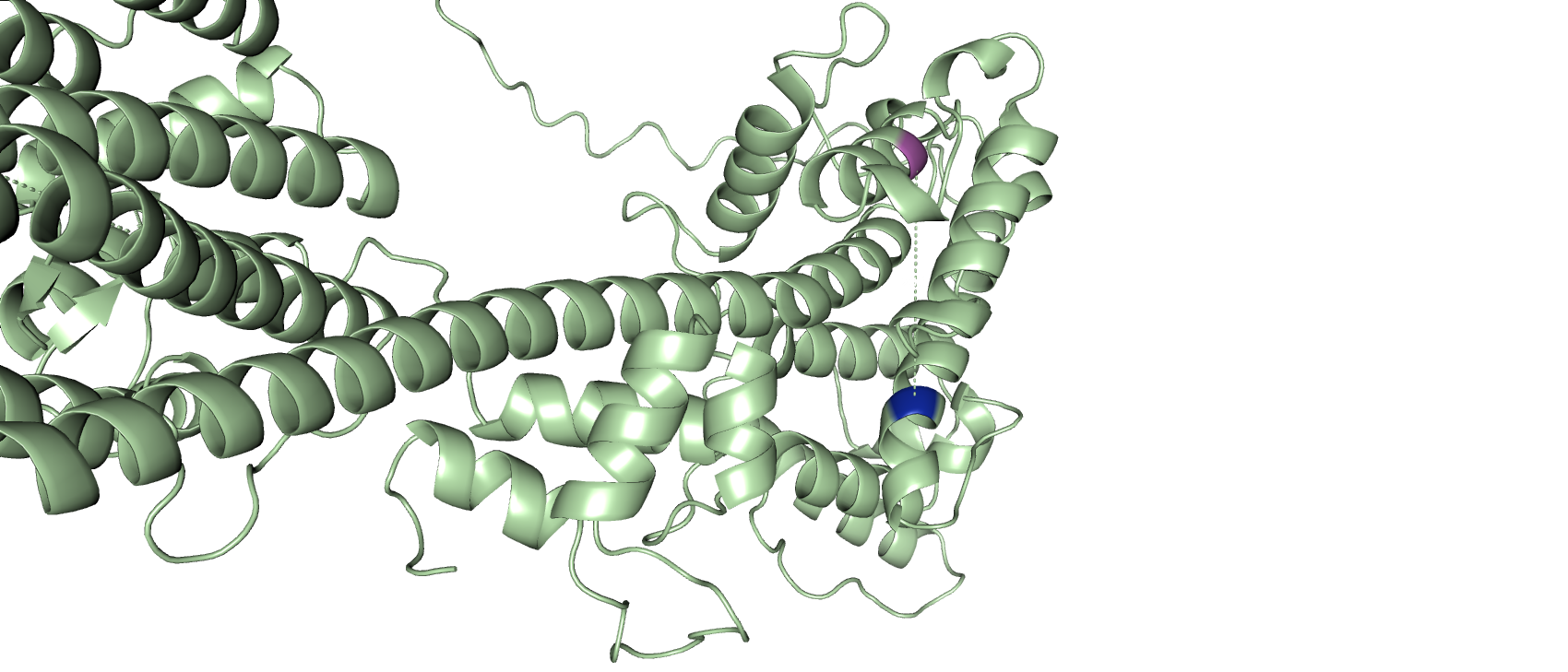
*

20.9Å

**Supplementary Figure S1:** Detail of the t-Cys and p-Ser sites in ACTN2 and the distance between both modified residues in the tertiary structure of the protein. Distance is represented in red and expressed in Angstrom (Å).

$$TPS index= -\log_{10} \left( \frac{\frac{ABS\left( RSMD tCys-RSMD pSer \right)}{RSMD tCys}+ \frac{ABS\left( RSMD traject tCys-RSMD traject pSer \right)}{RSMD traject tCys}}{2} \right)$$

**Supplementary Figure S2**: t-Cys vs. p-Ser similarity index (TPSi). This index reflects the similarity between the structure of t-Cys and p-Ser surrounding protein area. ABS is the absolute value of the difference between the values of the RMSD of t-Cys and p-Ser obtained by using 21-aminoacids fragments containing the specific p-Ser or t-Cys site ±10 amino acids from the PTM. Corresponding fragments with no modification were used as reference for the RMSD calculation of cysteic actid and phosphoserine substitution (RSMD tCys and RSMD pSer respectively). The RSMD trajectory (traject) values were obtained performing MD simulation of the fragments with cysteic acid and phosphoserine substitution.

**
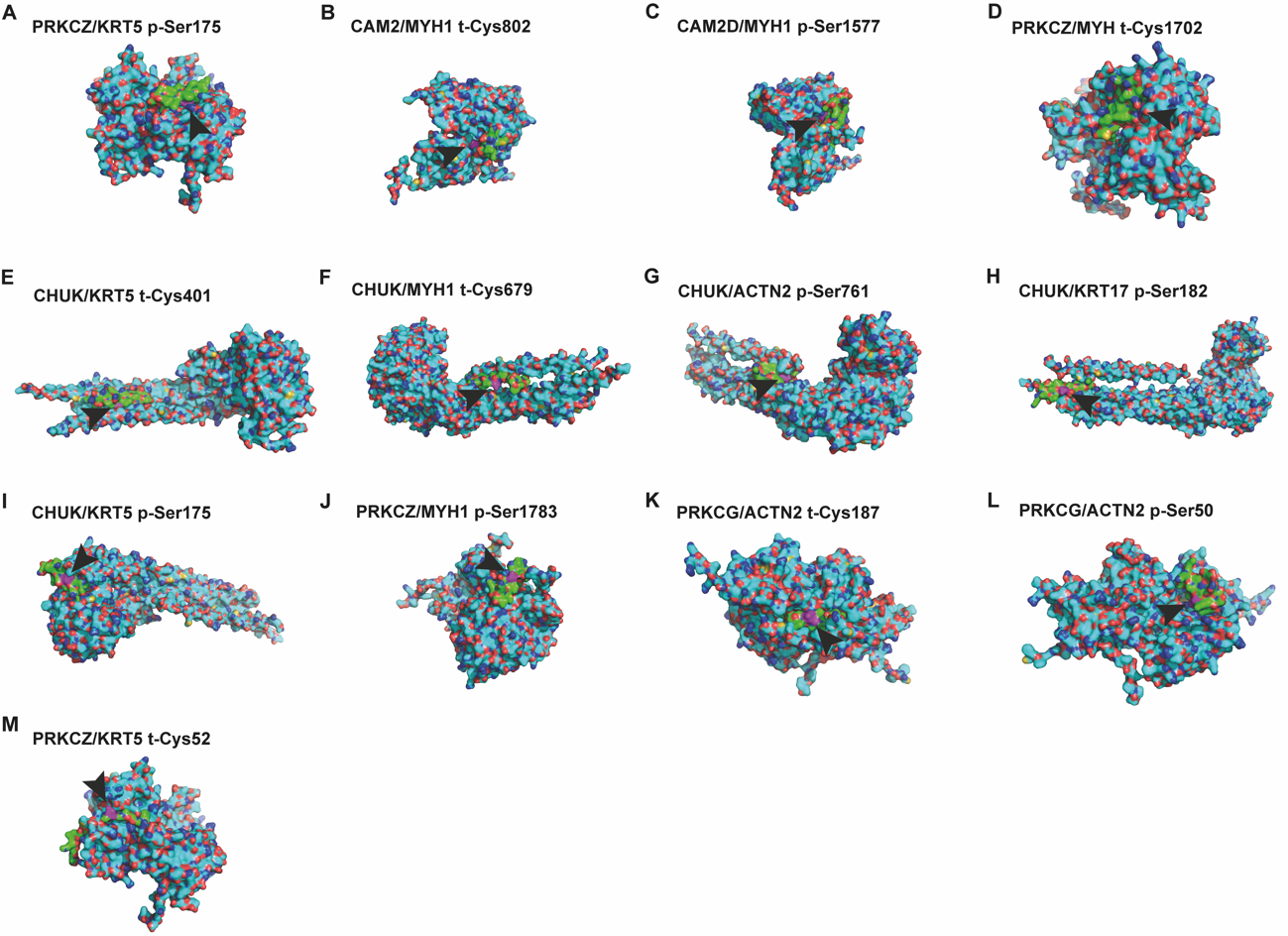
Supplementary Figure S3:** Docking simulation of the predicted kinases with the t-Cys and p-Ser sites. **A-M**. Docking models with the highest docking score values are displayed. Backbone of the kinase is shown in blue, backbone of the PTM containing peptide is shown in green and the t-Cys or p-Ser site is highlighted in magenta. For every structure, gene symbol of the kinase/gene symbol of the protein, residue position and PTM type are detailed.


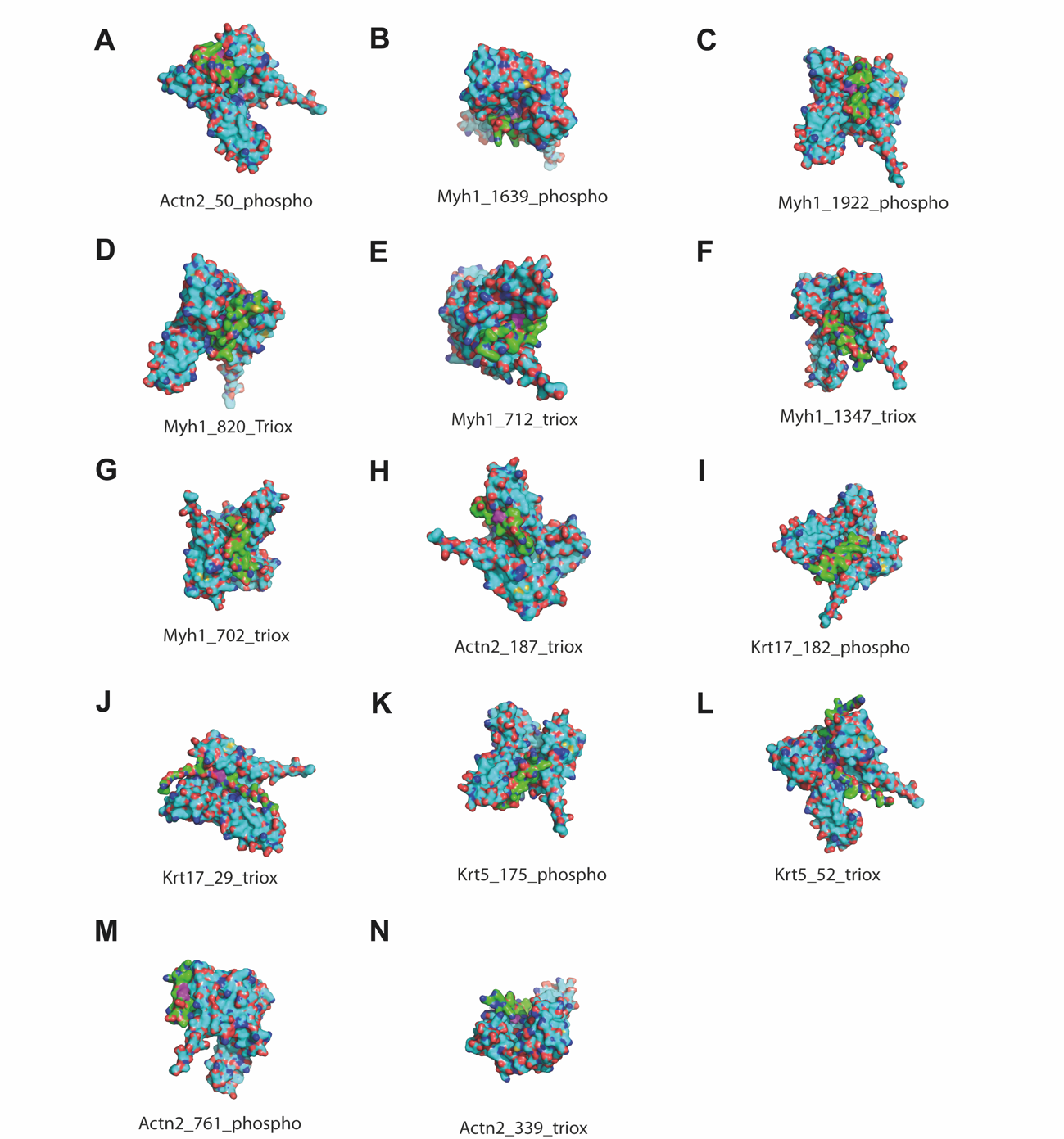


**Supplementary Figure S4:** Docking simulation of the 14-3-3σ protein with the t-Cys and p-Ser sites. **A-N**. Docking models with the highest docking score values are displayed. Backbone of the 14-3-3 protein σ is shown in blue, backbone of the PTM containing peptide is shown in green and the t-Cys or p-Ser site is highlighted in magenta. For every structure, gene symbol, residue position and PTM type are detailed.


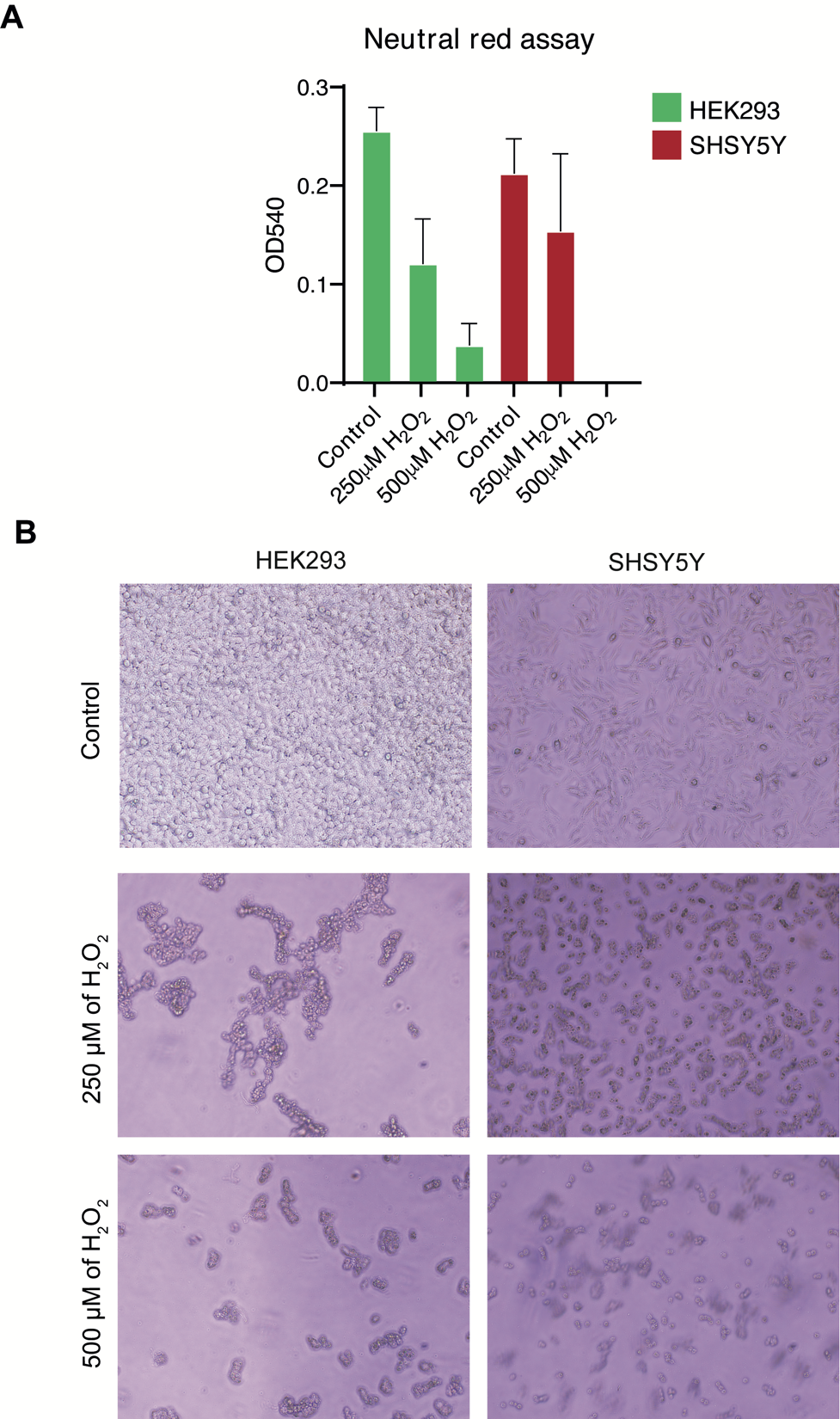


**Supplementary Figure S5:** Cellular viability of HEK293 and SHSY5Y cells under oxidative stress conditions. Oxidative stress was generated by addition of 250μM of H_2_O_2_ or 500 μM of H_2_O_2_. **A.** Neutral red uptake assay results of untreated HEK293 and SHSY5Y cells (Control) and cells treated with 250μM of H_2_O_2_ or 500 μM of H_2_O_2_ added into the medium. **B.** Optical microscopy micrographs (10X) of control HEK293 and SHSY5Y cells and cells treated with 250μM of H_2_O_2_ or 500 μM of H_2_O_2_.
